# Characteristics of Unknown Linear Systems Deduced from Measured CW Magnitude

**DOI:** 10.6028/jres.098.025

**Published:** 1993

**Authors:** M. T. Ma, J. W. Adams

**Affiliations:** National Institute of Standards and Technology, Boulder, CO 80303-3328

**Keywords:** Hilbert transform, impulse response, Laplace transform, linear system, minimum phase, system transfer function

## Abstract

A method is presented for predicting the total response, in both frequency and time, of an unknown linear system when only the measured continuous wave (cw) magnitude is available. The approach is based on approximating the square of the measured magnitude by a rational function, from which various system transfer functions in terms of complex frequency are deduced. These transfer functions may or may not be at minimum phase. The corresponding impulse response is then obtained by taking the inverse Laplace transform of the transfer function. The impulse response of the minimum-phase case rises faster initially to its first maximum than the nonminimum-phase counterparts. This result confirms that, for the same cw magnitude response, the accumulative energy contained in the impulse response is the greatest when the transfer function is at minimum phase. Physical meaning of the energy content is also discussed.

## 1. Introduction

The total response in frequency and time of a system to an assumed excitation, whether it is continuous wave (cw) or pulsed, is usually unpredictable if the system involves nonlinear elements. Even for linear but complex systems, the task of obtaining the total response when the system is excited by a source is still formidable because the complete system transfer function (amplitude and phase) may be unknown. The transfer function is defined as the ratio of system output to input in the frequency domain. The output and input can be voltage, current, electric field, magnetic field, or combinations of them. The time response of such a system required for assessing its vulnerability to an unfriendly electromagnetic environment can be determined only by sophisticated time-domain measurements or by derivations from the frequency-domain amplitude and phase measurements. Unfortunately, such time-domain measurements or frequency-domain phase measurements require expensive equipment and special considerations on radiation hazards, regulatory compliance, and environmental pollution (if performed outdoors). On the other hand, measuring magnitude response data of an unknown, complex, linear system to cw excitations at low levels, indoors or outdoors, is relatively straightforward, less costly, and free from compliance and pollution problems. Further, if such measured cw magnitude data can be processed to deduce a system transfer function, the phase characteristics and time response of such a system to a general excitation are then derivable.

In this paper, we present a method to deduce the total response of an unknown, complex, linear system from a given set of cw magnitude responses only. This is accomplished by approximating the square of the measured magnitude curve by a sum of ratios of two polynomials with real coefficients. Each of these ratios represents a second-order rational transfer function for a system with time-invariant lumped-constant elements. The exact number in the sum is determined by the number of resonant frequencies displayed in the measured magnitude data. Once the approximation work is done, the associated system transfer functions can then be obtained by using knowledge available from classical network theory. The transfer functions so obtained may or may not be at minimum phase. The corresponding phase characteristics and impulse responses are determined in a straightforward manner. For the same cw magnitude response, the accumulative energy associated with the impulse response corresponding to a minimum-phase transfer function is always greater than that corresponding to nonminimum-phase transfer functions. The derivations and detailed analysis are presented in Sec. 4.

The theoretical relationship between amplitude and phase of a system with a minimum-phase transfer function is outlined in Sec. 2. The conventional numerical approach for determining the phase characteristics and the corresponding time response of the linear minimum-phase system from the measured cw magnitude data, and the accuracy involved in this process are reviewed in Sec. 3. Examples are given in Sees. 4 and 5 to demonstrate the usefulness of the proposed method developed in Sec. 4. Energy contents associated with the given system are discussed in Sec. 6.

## 2. Theoretical Background

A stable linear system, however simple or complex, can be characterized by its transfer function *H*(*s*), which has no poles in the right half of the complex frequency s-plane. That is, *H*(*s*) is analytic in *Re*(*s*) ⩾ 0, where *Re* stands for the “real part of” [1]. We address only stable systems in this paper, because otherwise the system is not well designed, and therefore is not useful in application. In addition, when the system is made of only time-invariant and lumped-constant elements, its transfer function is then a rational function of *s* (a ratio of two polynomials with real coefficients) with the degree of the numerator polynomial lower than the degree of the denominator polynomial. When this transfer function is evaluated at *s* = *jω, H*(*jω*) is then a complex function of *ω*, consisting of a real part *R*(*ω*) and an imaginary part *X*(*ω*), or a magnitude |*H*(*jω*)| and a phase *θ*(*ω*). That is,

$$H(j\omega )=R(\omega )+jX(\omega )=|H(j\omega )|e^{-j\theta (\omega )},$$ (1)

where the convention of assigning a minus sign to the phase function is used. The magnitude function |*H*(*jω*)| may also be expressed in terms of the attenuation function *α*(*ω*):

$$|H(j\omega )|=e^{-\alpha (\omega )},\;\ln|H(j\omega )|=-\alpha (\omega ).$$ (2)

When *H*(*s*) is analytic as defined above and the system under study is causal [*h*(*t*) = 0 when *t* ⩽ 0], as is usually the case in practice, the real and imaginary parts of *H*(*jω*) are related by the Hilbert transform pair [2],

$$R(\omega )=\frac{2}{\pi }\int_{0}^{\infty }[yX(y)/(\omega^{2}-y^{2})]dy,$$ (3a)

and

$$X(\omega )=-\frac{2\omega }{\pi }\int_{0}^{\infty }[R(y)/(\omega^{2}-y^{2})]dy.$$ (3b)

In other words, the real and imaginary parts of this system are not independent. When one part is given either analytically or through measurement, the other part can be uniquely determined by performing one of integrals shown in Eq. (3). The complex transfer function *H*(*jω*) is then completely obtained, from which the impulse response may be derived. In reality, however, Eq. (3) is not useful because we cannot just measure the real or imaginary part of the system response to a given excitation.

If *H*(*s*), in addition to being analytic and causal, also has no zeros in the right half of the *s*-plane, the transfer function is said to be at minimum phase, herein denoted *by H*_m_(*s*). Under this condition, the attenuation function *α*(*ω*) and phase function *α*(*ω*) are related by another Hilbert transform pair [2,3,4],

$$\begin{array}{l} \theta (\omega )=\frac{\omega }{\pi }\int_{-\infty }^{\infty }[\alpha (y)/(y^{2}-\omega^{2})]dy\\ =-\frac{\omega }{\pi }\int_{-\infty }^{\infty }[\ln|H_{m}(jy)|/(y^{2}-\omega^{2})]dy, \end{array}$$ (4a)

and

$$\alpha (\omega )=\alpha (0)-\frac{\omega^{2}}{\pi }\int_{-\infty }^{\infty }[\theta (y)/y(y^{2}-\omega^{2})]dy.$$ (4b)

From Eq. (4b), we see that the attenuation function can be determined completely from a given phase function only when *α*(0) is also known. But, for our application, only Eq. (4a) is required because we assume that the magnitude (or attenuation) function is given by measurement. Once *θ*(*ω*) is determined from Eq. (4a), the entire complex *H*_m_(*jω*) can be obtained from Eq. (1) because | *H*_m_(*jω*)| is already given. The impulse response of this minimum-phase system *for t* ⩾ 0 is then calculated by the inverse Fourier transform.

$$h_{m}(t)=\frac{1}{2\pi }{\int_{-\infty }^{\infty }H_{m}(j\omega )e}^{j\omega t}dt.$$ (5)

The system’s time response to a general excitation can be computed by the convolution integral *h*_m_(*t*)*e(*t*), where *e*(*t*) represents an excitation, cw or pulse, applied to the system input. The success of determining the time response from magnitude data is based on the assumption that the system’s transfer function is at minimum phase. The solution of impulse response so obtained constitutes the only solution.

In general, however, there may be multiple solutions, because other possible transfer functions with nonminimum phases giving the same magnitude response may exist. One way of obtaining them with our proposed method is to be shown in Sec. 4.

## 3. Conventional Approach

Since the improper integral in Eq. (4a) is not easy to compute, the conventional approach has been to apply a transformation of variables known as the Wiener-Lee transform to *α*(*ω*) or |*H*(*jω*)| to obtain the necessary *θ*(*ω*). When the Wiener-Lee transform [2]

ω=−tan(δ/2) (6)

is applied, the integration interval (−∞, ∞) for *ω* in Eq. (4a) is transformed into (*−π, π*) for *δ.* The original attenuation function *α*(*ω*) and phase function *α*(*ω*) will be denoted, after transformation, respectively as *A* (*δ*) and *T*(*δ*). Since $\alpha (\omega )=-\text{ln}|H(j\omega )|=-\frac{1}{2}\ln[R^{2}(\omega )+X^{2}(\omega )]$ is an even function of *ω* and *θ*(*ω*) = −tan^−1^[*X*(*ω*)/*R*(*ω*)] is an odd function of *ω*, their respective transforms *A* (*δ*) and *T*(*δ*) will be even and odd functions of *δ*. As such, they may be expanded into Fourier cosine and sine series,

$$A(\delta )=a_{0}+a_{1}\cos\delta +a_{2}\cos2\delta +\ldots +a_{n}\cos n\delta +\ldots ,$$ (7)

and

$$T(\delta )=b_{1}\sin\delta +b_{2}\sin2\delta +\ldots +b_{n}\sin n\delta +\ldots ,$$

where the expansion coefficients are determined by

$$a_{0}=\frac{1}{2\pi }\int_{-\pi }^{\pi }A(\delta )d\delta =\frac{1}{\pi }\int_{0}^{\pi }A(\delta )d\delta ,$$ (8a)

$$a_{n}=\frac{1}{\pi }\int_{-\pi }^{\pi }A(\delta )\cos n\delta d\delta =\frac{2}{\pi }\int_{0}^{\pi }A(\delta )\cos n\delta d\delta ,$$

and

$$b_{n}=\frac{1}{\pi }\int_{-\pi }^{\pi }T(\delta )\sin n\delta d\delta =\frac{2}{\pi }\int_{0}^{\pi }T(\delta )\sin n\delta d\delta .$$ (8b)

When the system under consideration is causal, the expansion coefficients in Eq. (8) are simply related by [2]

$$b_{n}=-a_{n}.$$ (9)

Thus, when *α*(*ω*) or |*H*(*jω*)| is given, *A*(*δ*) is known. Determination of *a_n_* from Eq. (8a) automatically yields *b_n_* from Eq. (9), which in turn gives *T*(*δ*) and *θ*(*ω*) by means of Eq. (6), and hence the complex transfer function *H*(*jω*). The impulse response is then obtained from Eq. (5).

The justification for using the Wiener-Lee transform and the procedures as outlined above seem straightforward. The transform succeeds in converting the original improper integral in Eq. (4a) to a proper integral in Eq. (8a). From the application point of view, the important question is then: if the integral in Eq. (4a) is difficult to compute before the Wiener-Lee transform is applied, is it easier to compute *a_n_* in Eq. (8a) after the Wiener-Lee transform is used? The answer is most likely negative, because the integrand in Eq. (8a) involves complicated transcendental functions. This explains why, in practice, numerical computations are required. The entire procedure will then involve: (i) conversion of the measured data of *α*(*ω*) or |*H*(*jω*)| into *A*(*δ*) by Eq, (6), (ii) numerical calculation of *a_n_* from *A*(5) by Eq. (8a), (iii) construction of a transformed phase function *T*(*δ*) by including only a finite number of terms in the Fourier sine series with *b_n_ = −a_n_*, (iv) conversion of *T*(*δ*) back to *θ*(*ω*), (v) determination of the complex transfer function *H*(*jω*) based on the given *α*(*ω*) and the newly constructed *θ*(*ω*), and (vi) numerical computation of *h*(*t*) by Eq. (5). The numerical solution so obtained constitutes the only answer. Other possible solutions with nonminimum phases can never be found. In addition, each of the above six steps is an approximation, thus exerting doubt about the accuracy in the final solution [5, 6].

Thus, while the Hilbert transform is useful for processing measured cw data directly [7], it may not always offer advantage, together with the Wiener-Lee transform, for deriving the minimum phase.

## 4. Alternative but Simpler Approach

Using passive network theory [8, 9], we can deduce a rational transfer function *H*(*s*) directly and exactly from a squared magnitude function |*H*(*jω*)|^2^ expressed as a ratio of two polynomials of even order in *ω*, where the order of the numerator polynomial is at least two degrees lower than that of the denominator polynomial. Thus, if an approximate squared magnitude function |*H*(*jω*)|^2^ in such a form can be obtained from the measured cw magnitude data of an unknown, complex, linear system to some excitation, the task of deducing a rational transfer function, and subsequently, the associated phase function and impulse response (in time) is then straightforward. We will show later that multiple solutions for systems with the same |*H*(*jω*)| are possible. The transfer functions so deduced may or may not be at minimum phase. In this process, we essentially have assumed that the original unknown linear system, which may consist of distributed elements and other complexities, can be approximated by an equivalent passive network system with only time-invariant and lumped-constant elements. The approximation is the only one involved in the process. The exact order in the final approximate |*H*(*jω*)|^2^ depends on outstanding features in the given cw magnitude data. The most important feature displaying a strong resonance at a particular frequency can be approximated by a simple second-order transfer function.

### 4.1 Second-Order Transfer Function

The second-order transfer function may take either of the following two forms:

$$H_{2a}=A/(s^{2}+as+b),$$ (10a)

or

$$H_{2b}(s)=A(s+c)/(s^{2}+as+b),$$ (10b)

where the parameters *A*, *a, b*, and *c* are all real. In addition, we require

$$0<a<2\sqrt{b},$$ (11)

so that the complex poles are in the left half of the *s*-plane. On the other hand, the parameter *c* in Eq. (10b) may be positive, negative, or 0. When *c* is positive, the zero of the transfer function is also in the left half-plane (in fact, on the negative real axis). In this case, the transfer function is at minimum phase. When *c* is negative, the zero is in the right half-plane, and the transfer function is at nonminimum phase. When *c* = 0, the zero is at the origin, also constituting a nonminimum-phase case, and the dc magnitude response at *ω* = 0 is 0. The parameter *A* is used to match the given maximum magnitude response at the resonant frequency.

The outstanding features associated with the second-order transfer functions in Eq. (10) are examined in the following analysis.

#### 4.1.1 Second-Order Transfer Function in the Form of Eq. (10a)

In this case, we have

$$H_{2a}(j\omega )=A/(b-\omega^{2}+j\omega a).$$ (12a)

The squared magnitude is given by

$$\begin{array}{l} {\left|H_{2a}(j\omega )\right|}^{2}=H_{2a}(j\omega )H_{2a}(-j\omega )=H_{2a}(s)H_{2a}(-s)|s=j\omega \\ =\frac{A}{{\left(b-\omega^{2}\right)}^{2}+a^{2}\omega^{2}}=\frac{A^{2}}{\omega^{4}-(2b-a^{2})\omega^{2}+b^{2}}, \end{array}$$ (12b)

where *ω* is the only variable.

Setting the derivative of this squared magnitude to 0 yields *ω* = 0 and *ω*^2^ = (*2b* –*a*^2^)/2. If *b>a*^2^/2, *ω* = 0 gives the location of the minimum, while *ω*^2^ = (*2b* − *a*^2^)/2 gives the location of the maximum representing the location of the resonant frequency, herein designated as

$$\omega_{0}^{2}=(2b-a^{2})/2>0.$$ (13)

On the other hand, if a^2^/4< *b* <*a*^2^/2, |*H*(0)|^2^ will be the maximum. In this case, there is no resonant frequency. Thus, if a given magnitude curve has a resonant frequency at *ω*_0_ (other than 0), we require

$$b>a^{2}/2,$$ (14)

which is a stronger condition than that in Eq. (11).

In terms of *ω*_0_, the squared magnitude can be written as

$${\left|H_{2a}(j\omega )\right|}^{2}=\frac{A}{\omega^{4}-2\omega_{0}^{2}\omega^{2}+b^{2}}.$$ (15)

We then obtain the maximum

$${\left|H_{2a}(j\omega_{0})\right|}^{2}=\frac{A^{2}}{b^{2}-\omega_{0}^{4}},$$ (16)

which is also nonnegative because of Eq. (13), where $b=\omega_{0}^{2}+\frac{1}{2}a^{2}>\omega_{0}^{2}$.

The relative minimum at *ω* = 0 is given by

$$\left|H_{2a}(0)\right|=A/b.$$ (17)

When *ω* → ∞, |*H*_2_*_a_*(*jω*)| →0.

The half-power points may be defined as the frequencies at which the magnitude response of a linear system decreases to $\left(1/\sqrt{2}\right)$ of the peak response. The width between these frequencies represents a measure of sharpness of the magnitude response near the resonance. These frequencies are determined by

$${\left|H_{2a}(j\omega )\right|}^{2}=\frac{1}{2}{\left|H_{2a}(j\omega_{0})\right|}^{2}.$$ (18)

or

$$\omega^{4}-2\omega_{0}^{2}\omega^{2}+b^{2}=2(b^{2}-\omega_{0}^{4}),$$ (19)

which yields

$$\omega^{2}=\omega_{0}^{2}\pm \sqrt{b^{2}-\omega_{0}^{4}}.$$ (20)

If we denote the half-power frequency on the left side of the resonant frequency by *ω*_1_ and that on the right side by *ω*_2_, we have

$$\omega_{2}^{2}=\omega_{0}^{2}+\sqrt{b^{2}-\omega_{0}^{4}},\;\omega_{1}^{2}=\omega_{0}^{2}-\sqrt{b^{2}-\omega_{0}^{4}},$$ (21)

$\omega_{2}^{2}-\omega_{1}^{2}=2\sqrt{b^{2}-\omega_{0}^{4}}$, $\omega_{2}^{2}+\omega_{1}^{2}=2\omega_{0}^{2}$, and $\omega_{1}^{2}\omega_{2}^{2}=2\omega_{0}^{4}-b^{2}.$Clearly, $\omega_{1}^{2}$ and $\omega_{2}^{2}$ are symmetric with respect to $\omega_{0}^{2}$.

The quality factor of the system may be defined

$$Q=\omega_{0}/(\omega_{2}-\omega_{1}),$$ (22)

where *ω*_2_ − *ω*_1_ may be called bandwidth of the system.

Mathematically, we need three conditions to determine the three parameters *A*, *a*, and *b.* From the application point of view, we can express *A, a*, and *b* in terms of *ω*_0_, *ω*_1_, *ω*_2_, and |*H*(*jω*_0_)| from Eqs. (13), (16), and (21):

$$\begin{array}{l} A=(\omega_{2}^{2}-\omega_{1}^{2})|H_{2a}(j\omega_{0})|/2,\\ b^{2}=\omega_{0}^{4}+\frac{1}{4}{\left(\omega_{2}^{2}-\omega_{1}^{2}\right)}^{2}, \end{array}$$ (23)

and

$$a^{2}=2[\sqrt{\omega_{0}^{4}+{\left(\omega_{2}-\omega_{1}\right)}^{2}{\left(\omega_{2}+\omega_{1}\right)}^{2}/4}-\omega_{0}^{2}].$$

**Fig. 1:**
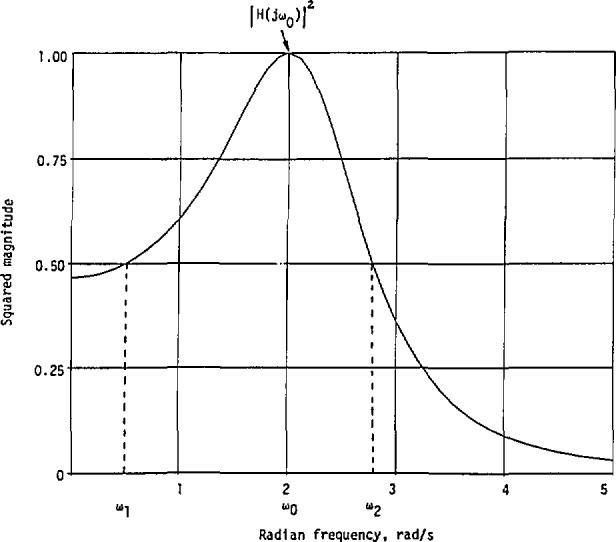
A squared-magnitude response with only one resonant frequency.

Thus, when there is only one resonant frequency in the measured cw magnitude curve for an unknown linear system, such as that shown in Fig. 1, the special features such as *ω*_0_, *ω*_1_, *ω*_2_, and |*H*(*jω*_0_)| can be read from it. The required parameters *A*, *a*, and *b* can then be determined from Eq. (23), regardless of whether the relation $\omega_{1}^{2}+\omega_{2}^{2}=2\omega_{0}^{2}$ is satisfied. The square of the given magnitude may be approximately represented by Eq. (12b), and the unknown linear system may be represented by the second-order transfer function Eq. (10a),

$$H_{2a}(s)=A/(s^{2}+as+b)=\frac{A}{{\left(s+a/2\right)}^{2}+\beta^{2}},$$ (24)

where

$$\beta^{2}=b-a^{2}/4.$$ (25)

Once this is done, we then obtain the associated phase function in accordance with the convention used in (1):

$$\begin{array}{c} \theta_{2a}(\omega )=\frac{1}{2j}\arg[H_{2a}(-j\omega )/H_{2a}(j\omega )]\\ =\tan^{-1}[a\omega /(b-\omega^{2})]. \end{array}$$ (26)

Since *a* and *b* are positive, *θ*_2_*_a_*(*ω*) varies from 0 to *π* when *ω* varies from 0 to ∞.

The corresponding impulse response can be determined by taking the inverse Laplace transform [10] of *H*_2_*_a_*(*s*) in Eq. (24),

$$h_{2a}(t)=\frac{A}{\beta }e^{-at/2}\sin\beta t,\;t⩾0.$$ (27)

The time response *R*(*t*) of this unknown linear system to a general excitation *e*(*t*), cw or pulse, is then given by the convolution integral [10],

$$R(t)=e(t)*h_{2a}(t).$$ (28)

If indeed the given squared magnitude curve with only one resonant frequency shown in Fig. 1 is represented by Eq. (12b) and the transfer function is represented by Eq. (24), the system under consideration will be at minimum phase, because this transfer function has no zero in the right half of the *s*-plane. In general, however, the system may also be at nonminimum phase because the actual transfer function could be represented by a product of *H*_2_*_a_*(*s*) and an all-pass function. In this case, the system transfer function with a nonmininium phase is given by

$$H_{n}(s)=H_{2a}(s)H_{\text{all}}(s),$$ (29)

with the simplest (first order) all-pass function defined as

$$H_{\text{all}}(s)=(s-\alpha )/(s+\alpha ),$$ (30)

where *α* is a real and positive number.

Since |*H*_all_(*jω*)|^2^ = 1, we have

$${\left|H_{n}(j\omega )\right|}^{2}={\left|H_{2a}(j\omega )\right|}^{2}{\left|H_{\text{all}}(j\omega )\right|}^{2}={\left|H_{2a}(j\omega )\right|}^{2}.$$ (31)

This implies that the same squared magnitude curve as that shown in Fig. 1 can be represented by either *H*_2_*_a_*(*s*) or *H*_n_(*s*).

In Eq. (30), the zero of *H*_all_(*s*) in the right half of the *s*-plane is the mirror image of the pole in the left half of the *s*-plane [8]. In general, the all-pass function may be of higher order with more zeros in the right half of the *s*-plane and the same number of mirror-image poles in the left half of the *s*-plane. These zeros are not necessarily limited to the real axis. They can take complex-conjugate pairs. If we restrict ourself, for the time being, to the first-order all-pass function given in Eq. (30), we can make further analysis. The impulse response for this nonminimum-phase transfer function may be determined from Eq. (29) by convolution integral [11],

$$\begin{array}{l} h_{n}(t)=h_{2a}(t)*{£}^{-1}[1-\frac{2\alpha }{s+\alpha }]\\ \;=h_{2a}(t)-2\alpha h_{2a}(t)*e^{-at}, \end{array}$$ (32)

where *£*^−1^ is the inverse Laplace transform.

The impulse response can also be obtained by taking partial fractions of Eq. (29). That is,

$$\begin{array}{c} H_{n}(s)=\frac{A(s-\alpha )}{\left(s^{2}+as+b)(s+\alpha \right)}\\ =A\left\{\frac{ps+q}{s^{2}+as+b}-\frac{p}{s+\alpha }\right\}\\ =A\left\{\frac{p(s+a/2)+q-ap/2}{{\left(s+a/2\right)}^{2}+\beta^{2}}-\frac{p}{s+\alpha }\right\}, \end{array}$$ (33)

where

$$\begin{array}{l} p=2\alpha /(b+\alpha^{2}-a\alpha ),\;\text{and}\\ q=(b-\alpha^{2}+a\alpha )/(b+\alpha^{2}-a\alpha ). \end{array}$$ (34)

The impulse response is then

$$h_{n}(t)=A\left\{e^{-at/2}[p\cos\beta t+\frac{q-ap/2}{\beta }\sin\beta t]-pe^{-at}\right\},$$ (35)

which is the same as Eq. (32) after the convolution is performed.

Both the minimum-phase impulse response *h*_2_*_a_*(*t*) in Eq. (27) and nonminimum-phase impulse response *h*_n_(*t*) in Eq. (35) vanish at *t* = 0. This can be confirmed by the initial-value theorem [10]. More detailed behaviors of Eqs. (27) and (35) can be learned by examining their first time derivatives,

$$h_{2a}^{\prime }(t)=\frac{A}{\beta }e^{-at/2}[\beta \cos\beta t-\frac{a}{2}\sin\beta t],$$ (36a)

and

$$h_{n}^{\prime }(t)=A\left\{(q-ap)\cos\beta t-\left[\frac{a(2q-ap)}{4\beta }+p\beta \right]\sin\beta t\right\}e^{-at/2}+Ap\alpha e^{-at}$$ (36b)

Clearly, at *t* = 0, $h_{2a}^{\prime }(0)=h_{n}^{\prime }(0)=A$. This means that both *h*_2_*_a_*(*t*) and *h*_n_(*t*) arise from 0 with the same starting rate. However, shortly afterward, they increase with different rates. At *t = ϵ*(1» *ϵ* >0), we may expand those functions involved and keep the first-order term to obtain

$$h_{2a}^{\prime }(\epsilon )\approx A{\left(1-a\epsilon /2\right)}^{2}\approx A(1-a\epsilon )$$ (37a)

and

$$h_{n}^{\prime }(\epsilon )\approx A[1-(a+\alpha )\epsilon ].$$ (37b)

Since both *a* and *α* are real and positive, we conclude that

$$h_{n}^{\prime }(\epsilon )<h_{2a}^{\prime }(\epsilon ).$$ (38a)

By similar steps we can also show that

$$h_{n}(\epsilon )<h_{2a}(\epsilon ).$$ (38b)

The relations in Eqs. (38a) and (38b) imply that shortly after the system is excited by a source, *h*_2_*_a_*(*t*) associated with the minimum-phase system increases with a greater rate than the nonminimum-phase counterpart *h*_n_(*t*). Although this point is drawn from a special case (first-order all-pass function), it can be generalized to higher orders even though the algebraic derivations are much more involved. Equations (38a) and (38b) also mean that more energy accepted by the minimum-phase system is concentrated at the beginning (*t* = 0+) of the excitation than the nonminimum-phase system [7, 12]. Details on energy consideration are found in Sec. 6. This observation is very important from the standpoint of electromagnetic interferences (EMI). If the minimum-phase system can survive the initial impact due to an unwanted external source, a nonminimum-phase system can also survive it. The minimum-phase system may be considered the worst case as far as the initial impact due to an unwanted signal is concerned. From the design point of view, if a system is minimum phase, the designer may wish to convert it to nonminimum phase by adding an all-pass network to reduce initial EMI impact.

From Eq. (36a) we know that the first maximum of *h*_2_*_a_*(*t*) occurs at *t*_m0_, which is the smallest root of

tanβt=2β/a. (39)

After reaching its first peak at *t*_m0_, the impulse response *h*_2_*_a_*(*t*) varies sinusoidally with a decay rate of *a/2* and with a period of *β*. Although it is not as straightforward to determine the exact location of the first maximum for *h*_n_(*t*) by setting Eq. (36b) to 0, we know that it also varies sinusoidally with the same period *β* but decays with a different rate because of the extra term exp(−*αt*). A numerical example is here presented to illustrate this point.

Example 1. Suppose that the square of a “measured” cw magnitude curve can be represented by

$$f^{2}(\omega )=e^{-{\left(\omega^{2}-4\right)}^{2}}$$ (40)

For this example, the resonant frequency occurs at *ω*_0_ = 2. The half-power frequencies are $\omega_{1}^{2}=3.1674$, and $\omega_{2}^{2}=4.8326$. The bandwidth is given by *ω*_2_−*ω*_1_ = 0.4185, and *Q*=4.7780. Since $\omega_{1}^{2}+\omega_{2}^{2}=2\omega_{0}^{2}$, we can use *H*_2_*_a_*(*s*). The required parameters can be obtained from Eq. (23) as: *a* = 0.4141, *b* = 4.0857, and *A* = 0.8326 (carried to 4 digits). The approximate squared magnitude is then

$${\left|H_{2a}(j\omega )\right|}^{2}=\frac{0.6931}{\omega^{4}-8\omega^{2}+16.6931}.$$ (41)

By presenting the numerical results in Table 1, we see that the given curve in Eq. (40) and its approximation in Eq. (41) indeed match at *ω*_1_, *ω*_2_, and *ω*_0_. After obtaining Eq. (41), we have the following simplest solution to represent the transfer function with a minimum phase:

$$H_{2a}(s)=0.8326/(s^{2}+0.4141s+4.0857).$$ (42)

The associated phase function and impulse response are then respectively

$$\begin{array}{l} \theta_{m}(\omega )=\tan^{-1}[a\omega /(b-\omega^{2})]\\ \;=\tan^{-1}[0.4141\omega /(4.0857-\omega^{2})] \end{array}$$ (43)

and

$$h_{2a}(t)=0.4141e^{-0.2070t}\sin(2.0107t),\;t⩾0.$$ (44)

For the same |*H*_2_*_a_*(*jω*)|^2^ obtained in Eq. (41), we could also have a transfer function with a non- minimum phase by including an all-pass function, say, the first order with *α* = 1. We then have

$$H_{n}(s)=\frac{0.8326(s-1)}{\left(s^{2}+0.4141s+4.0857)(s+1\right)}.$$ (45)

The corresponding phase function and impulse function (after taking partial fractions) are

$$\theta_{n}(\omega )=\theta_{m}(\omega )+\tan^{-1}(\omega )+\pi ,$$ (46)

with *θ*_m_(*ω*) given in Eq. (43) and

$$\begin{array}{l} h_{n}(t)=[0.3564\cos(2.0107t)+0.2735\\ \;\sin(2.0107t)]e^{-0.2070t}\\ \;-0.3564e^{-t},\;t⩾0. \end{array}$$ (47)

**Fig. 2:**
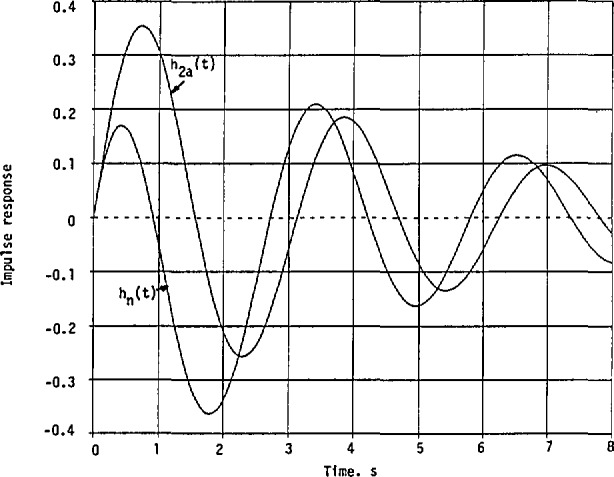
Impulse responses of the minimum-phase and nonminimum-phase systems with their given squared-magnitude cw response in Eq. (40) and the approximate squared magnitude in Eq. (41).

The impulse responses obtained in Eq. (44) and Eq. (47) are plotted in Fig. 2 to confirm the conclusions in Eqs. (38a) and (38b). Thus, more energy is concentrated in *h*_2_*_a_*(*t*) than in *h*_n_(*t*) at the beginning of excitation.

The solutions for *θ*_n_(*ω*) and *h*_n_(*t*) are not unique because they depend on the choice of specific all-pass functions.

We note from Eq. (23) that the parameter *a* decreases with the bandwidth (*ω*_2_ − *ω*_1_) or is inversely proportional to *Q*, and that the parameter *b* is primarily determined by $\omega_{0}^{2}$. These parame- ters decide respectively, in turn, the decay rate and period of variations of the impulse response.

#### 4.1.2 Second-Order Transfer Function Taking the Form of Eq. (10b)

For this case, we have

$$H_{2b}(j\omega )=\frac{A(c+j\omega )}{b-\omega^{2}+j\omega a}$$ (48)

and

$${\left|H_{2b}(j\omega )\right|}^{2}=\frac{A^{2}(c^{2}+\omega^{2})}{\omega^{4}-(2b-a^{2})\omega^{2}+b^{2}}.$$ (49)

Now there are three solutions for d|H_2_*_b_*(*jω*)|^2^/d*ω* = 0. One of these, *ω* = 0, is the minimum with |*H*(0)|^2^ = *A*^2^*c*^2^/*b*^2^. The other solution gives the resonant frequency,

$$\omega_{0}^{2}=-c^{2}+\sqrt{c^{4}+b^{2}+2bc^{2}-a^{2}c^{2}},$$ (50)

which is always greater than 0 (as it should be) in view of Eq. (14). A third solution takes the same form as Eq. (50) except with a negative sign in front of the square-root sign. This third solution is obviously nonphysical.

The maximum value of the squared magnitude at Wo is, after substitution of Eq. (50) into Eq. (49) and some algebraic simplification, given by

$${\left|H_{2b}(j\omega_{0})\right|}^{2}=A^{2}/(2\omega_{0}^{2}-2b+a^{2}).$$ (51)

Again, this is the only maximum. The general variation of |*H*_2_*_b_*(*jω*)|^2^ is similar to that in Fig. 1.

The half-power frequencies *ω*_1_ and *ω*_2_ are determined by

$$\frac{\omega^{2}+c^{2}}{\omega^{4}-(2b-a^{2})\omega^{2}+b^{2}}=\frac{1}{4(\omega_{0}^{2}-b)+2a^{2}}$$

or

$$\omega^{4}+(2b-a^{2}-4\omega_{0}^{2})\omega^{2}+b^{2}-c^{2}(4\omega_{0}^{2}+2a^{2}-4b)=0.$$ (52)

Instead of solving for *ω*_1_ and *ω*_2_, we note that

$$\omega_{1}^{2}+\omega_{2}^{2}=4\omega_{0}^{2}+a^{2}-2b,$$ (53)

and

$$\omega_{1}^{2}\omega_{2}^{2}=b^{2}-c^{2}(4\omega_{0}^{2}+2a^{2}-4b).$$ (54)

The above derivations represent analysis for the particular transfer function *H*_2_*_b_*(*s*). From the application viewpoint, we can express *a, b*, and c in terms of *ω*_0_, *ω*_1_, and *ω*_2_ by using Eqs. (50), (53), and (54),

$$\begin{array}{l} b^{2}=2\omega_{0}^{4}-\omega_{1}^{2}\omega_{2}^{2},\\ c^{2}=(\omega_{0}^{4}-\omega_{1}^{2}\omega_{2}^{2})/(\omega_{1}^{2}+\omega_{2}^{2}-2\omega_{0}^{2})\\ a^{2}=\omega_{1}^{2}+\omega_{2}^{2}-4\omega_{0}^{2}+2b. \end{array}$$ (55)

Thus, when *ω*_0_, *ω*_1_, and *ω*_2_ are read from a mea- sured magnitude curve, we can easily determine the required parameters *b, c*, and *a* from Eq. (55). Since *a*^2^, *b*^2^, and the denominator of *c*^2^ [which is equal to the denominator of Eq. (51)] are all positive, and *c*^2^ itself is nonnegative, we require

$$\omega_{1}^{2}+\omega_{2}^{2}>2\omega_{0}^{2}\;\text{and}\;\omega_{0}^{2}⩾\omega_{1}\omega_{2}.$$ (56)

In addition, the constant factor *A* can be determined from Eq. (51),

$$A^{2}=(2\omega_{0}^{2}-2b+a^{2}){\left|H(j\omega_{0})\right|}^{2},$$ (57)

where |*H*(*jω*_0_)|^2^ can also be obtained from the given magnitude curve.

For the special case *c* = 0 when $\omega_{0}^{2}=\omega_{1}\omega_{2}$, we have

$$\begin{array}{l} b=\omega_{0}^{2}=\omega_{1}\omega_{2},\\ a^{2}=\omega_{1}^{2}+\omega_{2}^{2}-2\omega_{0}^{2}=\omega_{1}^{2}+\omega_{2}^{2}-2\omega_{1}\omega_{2}={\left(\omega_{2}-\omega_{1}\right)}^{2},\\ {}[\text{or}\;a=\omega_{2}-\omega_{1}], \end{array}$$ (58)

and

$$A^{2}=a^{2}{\left|H(j\omega_{0})\right|}^{2},\;or\;A=a\left|H(j\omega_{0})\right|.$$

Here, the parameter *a* is controlled solely by the system’s bandwidth, and *b* depends only on the resonant frequency. Also, for this special case, *ω*_0_ is the geometrical mean of *ω*_1_ and *ω*_2_.

One unique feature associated with this case [Eq. (10b)] is that once an approximate squared magnitude in the form of Eq. (49) is obtained from the given magnitude curve by the procedures thus outlined, the solutions for the transfer function are not unique. One of the obvious solutions, herein designated as *H_m_(s)* takes the same expression given in Eq. (10b),

$$H_{m}(s)=\frac{A(s+c)}{s^{2}+as+b},$$ (59)

where *c* is taken as a positive number from Eq. (55). In this case, the transfer function is in minimum phase.

The other solution is

$$H_{n}(s)=\frac{A(c-s)}{s^{2}+as+b},$$ (60)

which represents a nonminimum-phase transfer function.

Clearly, we have |*H*_m_(*jω*)|^2^ = |*H*_n_(*jω*)|^2^.

The associated phase function for *H*_m_(*s*) can be obtained directly from *H*_m_(*jω*) as

$$\theta_{m}(\omega )=\tan^{-1}[a\omega /(b-\omega^{2})]-\tan^{-1}(\omega /c).$$ (61)

The second term becomes *π*/2 when *c* = 0. Since the first term varies from 0 to *π* and the second term varies from 0 to *π*/2 as *ω* changes from 0 to ∞, the range of variation for *θ*_m_(*ω*) is hence from 0 to *π*/2.

The phase function for *H*_n_(*s*) is

$$\theta_{n}(\omega )=\tan^{-1}[a\omega /(b-\omega^{2})]+\tan^{-1}(\omega /c),$$ (62)

whose range of variation is from 0 to 3*π*/2.

According to the Hilbert transform given in Eq. (4a), the phase function *θ*_m_(*ω*) may also be obtained from the deduced squared magnitude,

$$\begin{array}{c} \theta_{m}(\omega )=-\frac{\omega }{\pi }\int_{-\infty }^{\infty }[\ln\left|H_{m}(jy)\right|/(y^{2}-\omega^{2})]\mathrm{dy}\\ =-\frac{\omega }{\pi }\int_{-\infty }^{\infty }\frac{\ln(y^{2}+c^{2})\mathrm{dy}}{2(y^{2}-\omega^{2})}\\ +\frac{\omega }{\pi }\int_{-\infty }^{\infty }\frac{\ln[y^{4}-(2b-a^{2})y^{2}+b^{2}]\mathrm{dy}}{2(y^{2}-\omega^{2})} \end{array}$$ (63)

Because the integrands are even functions of *y*, we have

$$\begin{array}{c} \theta_{m}(\omega )=-\frac{\omega }{\pi }\int_{0}^{\infty }\frac{\ln(y^{2}+c^{2})\mathrm{dy}}{y^{2}-\omega^{2}}\\ +\frac{\omega }{\pi }\int_{0}^{\infty }\frac{\ln(y^{2}+m^{2})\mathrm{dy}}{y^{2}-\omega^{2}}\\ +\frac{\omega }{\pi }\int_{0}^{\infty }\frac{\ln(y^{2}+n^{2})\mathrm{dy}}{y^{2}-\omega^{2}}, \end{array}$$ (64)

where we have broken the last integral in Eq. (63) into two parts with

$$m^{2}+n^{2}=-2b+a^{2},\;\text{and}\;m^{2}n^{2}=b^{2}.$$ (65)

Since a known definite integral in the form of Eq. (64) is available [13],

$$\begin{array}{l} \int_{0}^{\infty }\frac{\ln(f^{2}+g^{2}x^{2})dx}{h^{2}x^{2}-k^{2}}\\ =\frac{\pi }{hk}\tan^{-1}(gk/fh),\;f,g,h,k>0, \end{array}$$ (66)

we easily, after comparing Eqs. (64) and (66), identify *g = h* = 1, *k = ω*, *f = c, m*, and *n* respectively for the first, second, and third integrals in Eq. (64), and thus obtain

$$\begin{array}{c} \theta_{m}(\omega )=-\tan^{-1}(\omega /c)+\tan^{-1}(\omega /m)+\tan^{-1}(\omega /n),\\ =-\tan^{-1}(\omega /c)+\tan^{-1}[a\omega /(b-\omega^{2}) \end{array}$$ (67)

which is identical to Eq. (61). The last step in Eq. (67) is accomplished by combining tan^−1^(*ω*/*m*) and tan^−1^(*ω*/*n*) after using the relations in Eq. (65) and noting the requirements in Eq. (66). The phase function for *H*_n_(*jω*), however, cannot be obtained from the Hilbert transform.

The derivation of Eq. (67) from |*H*_m_(*jω*)|^2^ is exact. In general, there is no fijrther approximation involved once an approximate squared magnitude in the form of a ratio of two polynomials in even orders of *ω* is deduced from a measured cw magnitude curve. In fact, the phase of the minimum-phase transfer function can be obtained directly from this deduced squared magnitude with the help of Eq. (4a) by the method proposed here. The type of integral formula given in Eq. (66) together with the Hilbert transform Eq. (4a) can also be applied to *H*_2_*_a_*(*s*) in Eq. (24) to obtain the same phase function *θ*_2_*_a_*(*ω*) in Eq. (26).

The corresponding impulse responses are determined from

$$H_{m}(s)=A\frac{\left(s+c\right)}{s^{4}+as+b}=A\frac{s+a/2+c-a/2}{{\left(s+a/2\right)}^{2}+\beta^{2}}$$ (68)

and

$$H_{n}(s)=A\frac{c+a/2-(s+a/2)}{{\left(s+a/2\right)}^{2}+\beta^{2}}$$ (69)

yielding respectively

$$h_{m}(t)=Ae^{-at/2}[\cos\beta t+\frac{c-a/2}{\beta }\sin\beta t],\;t⩾0,$$ (70)

and

$$h_{n}(t)=Ae^{-at/2}[\cos\beta t-\frac{c+a/2}{\beta }\sin\beta t],\;t⩾0,$$ (71)

where *β* is given in Eq. (25).

These impulse functions are still, basically, a sinusoidal function with a decay rate of *a/2*, even though the form is little more complicated than that of *h*_2_*_a_*(*t*) given in Eq. (27). Again, the decay rate is related primarily to the bandwidth, and the period of variations to the resonant frequency.

From Eqs. (70) and (71), we see that *h*_m_(0+) = *A*[= −*h*_n_(0+)], which is different from the previous case where *h*_2_*_a_*(0+) = 0. Both impulse responses start (*t* = 0) at the same magnitude *A.* Since the parameter *c* can be greater than *a*/*2*, at *t* = 0+, *h*_m_(*t*) may increase and reach the maximum before falling to its first zero. On the other hand, |*h*_n_(*t*)| always deaeases beginning at *t* = 0+. The first zero of *h*_m_(*t*) is determined by the smallest root of

$$\tan\beta t_{m}=-\beta /(c-a/2),$$ (72)

while that of *h*_n_(*t*) is by the smallest root of

$$\tan\beta t_{n}=-\beta /(c+a/2).$$ (73)

A comparison of Eqs. (72) with (73) indicates, regardless of the relative values of *c* and *a*/2, that the first zero of *h*_n_(*t*), called *t*_n0_, is no greater than that of *h*_m_(*t*) called *t*_m0_. That is,

$$t_{n0}⩽t_{m}0.$$ (74)

The equality sign holds when *c* = 0.

Equation (74) implies that *t*_n0_ is closer to *t* = 0 than is *t*_m0_. Thus, the beamwidth of the impulse response *h*_m_(*t*) associated with the minimum-phase transfer function is wider than the beamwidth of |*h*_n_(*t*)| associated with the nonminimum-phase transfer function with the same squared-magnitude function. This, in turn, means that more energy is concentrated, at the beginning of excitation, in the minimum-phase system than in the nonminimum-phase system. Once again, the minimum-phase system may be considered as the worst case as far as the initial impact of the system by the interference source is concerned. Another example is presented for illustration.

Example 2. Suppose that the square of a given “measured” magnitude can be represented by a shifted Gaussian function,

$$f^{2}(\omega )=4e^{-2(\omega -3)2}$$ (75)

The resonant and two half-power frequencies are: *ω*_0_ = 3, *ω*_1_ = 2.4113, and *ω*_2_ = 3.5887, the system bandwidth is 1.1774, and *Q* = 2.5480. The maximum at *ω*_0_ is 4. Since $\omega_{1}^{2}+\omega_{2}^{2}$ is not equal to $2\omega_{0}^{2}$ in this case, we wish to approximate Eq. (75) by |*H*_2_*_b_*(*jω*)|^2^ given in Eq. (49). Using Eq. (55) we obtain the required parameters: *a*^2^ = 1.3606, *b*^2^ = 87.1181, *c*^2^ = 8.8267, and *A*^2^ = 2.7725. Then

$${\left|H_{2b}(j\omega )\right|}^{2}=\frac{2.7725(\omega^{2}+8.8267)}{\omega^{4}-17.3069\omega^{2}+87.1181},$$ (76)

which is computed together with *f*^2^(*ω*) in Table 2 to show the quaUty of approximation.

The computation is carried out only from *ω* = 0 to *ω* = 5.50. The approximation around the important region near *ω* = *ω*_0_ = 3 is very good, while that near the two ends (*ω* = 0 and *ω* = 5.50) is marginal. Physically, however, the less accurate results near the two frequency ends are of secondary importance.

If we deal with |*H*_2_*_b_*(*jω*)|^2^ alone without including extra all-pass functions, we have two possible solutions for the transfer function, one with a minimum phase and the other with a nonminimum phase,

$$\begin{array}{l} H_{m}(s)=\frac{1.6651(s+2.9710)}{s^{2}+1.1664s+9.3337}\\ \;=\frac{1.6651(s+0.5832+2.3878)}{{\left(s+0.5832\right)}^{2}+2.9989^{2}} \end{array}$$ (77)

and

$$\begin{array}{l} H_{n}(s)=\frac{1.6651(2.9710-s)}{s^{2}+1.1664s+9.3337}\\ \;=\frac{1.6651[3.5542-(s+0.5832)]}{{\left(s+0.5832\right)}^{2}+2.9989^{2}}. \end{array}$$ (78)

The corresponding phases are

$$\begin{array}{c} \theta_{m}(\omega )=\tan^{-1}[1.1664\omega /(9.3337-\omega^{2})]\\ -\tan^{-1}(\omega /2.9710) \end{array}$$ (79)

and

$$\begin{array}{c} \theta_{n}(\omega )=\tan^{-1}[1.1664\omega /(9.3337-\omega^{2})]\\ +\tan^{-1}(\omega /2.9710) \end{array}$$ (80)

The impulse responses are

$$\begin{array}{l} h_{m}(t)=1.16651[\cos(2.9989t)\\ \;+0.7962\sin(2.9989t)]e^{-0.5832t} \end{array}$$ (81)

and

$$\begin{array}{l} h_{n}(t)=-1.16651[\cos(2.9989t)\\ \;-1.1852\sin(2.9989t)]e^{-0.5832t} \end{array}$$ (82)

Thus, this method not only yields a solution with a minimum-phase transfer function such as by the conventional numerical method described in Sec. 3, but also gives other possible solutions with nonminimum-phase transfer functions.

**Fig. 3:**
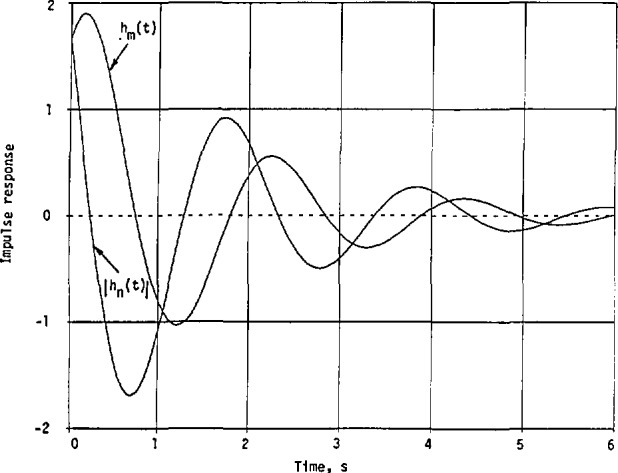
Impulse responses with the given cw squared-magnitude response in Eq. (75) and the approximate squared magnitude in Eq. (76).

Figure 3 shows *h*_m_(*t*) and |*h*_n_(*t*)| for comparison purpose. Evidently, both *h*_m_(*t*) and |*h*_n_(*t*)| begin at *t* = 0 with a magnitude of 1.6651. Then *h*_m_(*t*) increases to its maximum of 1.9030 at *t* = 0.1602, and *−h*_n_(*t*) starts to decrease. The first zero of *h*_n_(*t*) is at *t* = 0.2337, while that of *h*_m_(*t*) is at *t* = 0.7480. Also, |*h*_n_(*t*)|_max_ < |*h*_m_(*t*)|_max_ in this case. More energy is concentrated in *h*_m_(*t*) than in *h*_n_(*t*) near *t* = 0.

Comparing Eqs. (77) and (78), we may also express *H*_n_(*s*) in terms of *H*_m_(*s*) and a first-order all-pass function:

$$H_{n}(s)=H_{m}(s)\frac{2.9710-s}{2.9710+s}.$$ (83)

In general, the solution obtained in Eqs. (69) and (71) is not the only one with a nonminimum phase. In fact, the same given magnitude curve can also be represented by the product of *H*_m_(*s*) [or *H*_n_(*s*)] and additional all-pass functions. The solutions depend on the choice of these extra all-pass functions.

### 4.2 First-Order Transfer Function

The two types of the second-order transfer function analyzed in Sec. 4.1 are the most important ones which can be used to approximate a measured magnitude curve with only one resonant frequency not at *ω* = 0. For studies of radiated susceptibility, these may be sufficient because it is difficult or meaningless for an antenna to measure the interference response of linear systems at *ω* = 0. However, when the dc interference is also possible, in addition to the cw interferences, for some practical systems, the response at *ω* = 0 may constitute a relative maximum. To cover this case, we can approximate this part of the given measured magnitude curve by a squared magnitude corresponding to the first-order stable transfer function

$$H_{1}(s)=A/(s+a),$$ (84)

where *A* and *a* are real and positive. Its squared magnitude is

$${\left|H_{1}(j\omega )\right|}^{2}=A^{2}/(\omega^{2}+a^{2}).$$ (85)

**Fig. 4:**
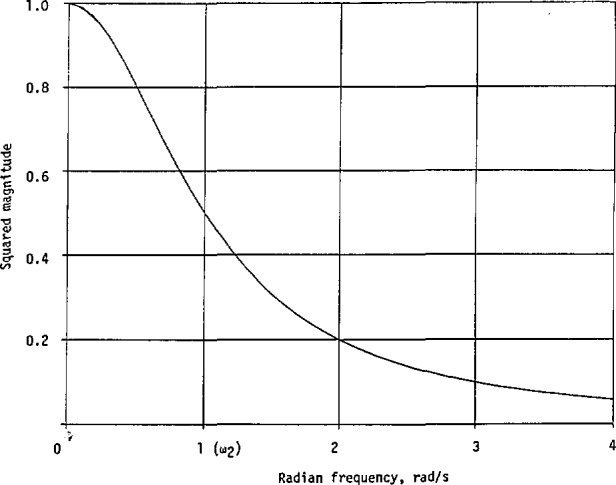
A squared-magnitude response corresponding to the first-order transfer function, normalized Eq. (85).

Obviously, its only maximum occurs at *ω* = 0, with |*H*_1_(0)|^2^ = (*A*/*a*)^2^. A representative curve for |*H*_1_(*jω*)|^2^ is shown in Fig. 4.

The half-power frequency may be determined by

$$1/(\omega^{2}+a^{2})=1/(2a^{2}),$$ (86)

which yields only one solution *ω*_2_ *= a* (the other half-power frequency *ω*_1_ *= −a* has no physical meaning). The bandwidth in this case is just 2*ω*_2_.

The associated phase function can be obtained either from *H*_1_(*jω*) or from the Hilbert transform,

$$\theta_{1}(\omega )=\tan^{-1}(\omega /a),$$ (87)

which varies from 0 to *π*/2 as *ω* varies from 0 to ∞.

The corresponding impulse response is

$$h_{1}(t)=Ae^{-at},\;t⩾0.$$ (88)

The decay rate in this case equals numerically the half-power frequency.

### 4.3 More General Case

For a more general case where the measured magnitude curve has *N*>1 distinct resonant frequencies, it may be approximated with our proposed method by a sum of terms of the type |*H*_2_*_a_*(*jω*)|^2^ or |*H*_2_*_b_*(*jω*)|^2^ discussed in Sec. 4.1. To simplify the notation, let us temporarily drop the subscript *a* and *b* while maintaining the subscript 2 to indicate the order of the transfer function being considered. The approximated squared magnitude will then take the form:

$${\left|H(j\omega )\right|}^{2}=\sum_{i=1}^{N}{\left|H_{2i}(j\omega )\right|}^{2},$$ (89)

with the required parameters *a, b, A*, and possibly *c* in each |*H*_2_(*jω*)|^2^ to be determined by the outstanding features associated with each resonant frequency. Should there also be a relative maximum at *ω* = 0, another term in the form of Eq. (85) for a first-order transfer function may be added to Eq. (89). Once this approximation is accomplished, the system transfer functions can then be deduced by the classical method [8]. We can then determine from these transfer functions the corresponding phase functions and impulse responses to give the complete characteristics of the unknown linear system. Another example is given below to illustrate this point.

Example 3. Suppose that the square of a measured cw magnitude curve can be represented by a sum of two mathematical expressions

$$f^{2}(\omega )=f_{1}^{2}(\omega )+f_{2}^{2}(\omega ),$$ (90)

where

$$f_{1}^{2}(\omega )=e^{-2\omega }$$ (91)

is used to simulate a possible maximum at *ω* = 0, and

$$f_{2}^{2}(\omega )=4e^{-2{\left(\omega -3\right)}^{2}}$$ (92)

is simulated for a possible resonant frequency at *ω*_0_ = 3. The expression in Eq. (92) is the same as that presented in example 2.

**Fig. 5:**
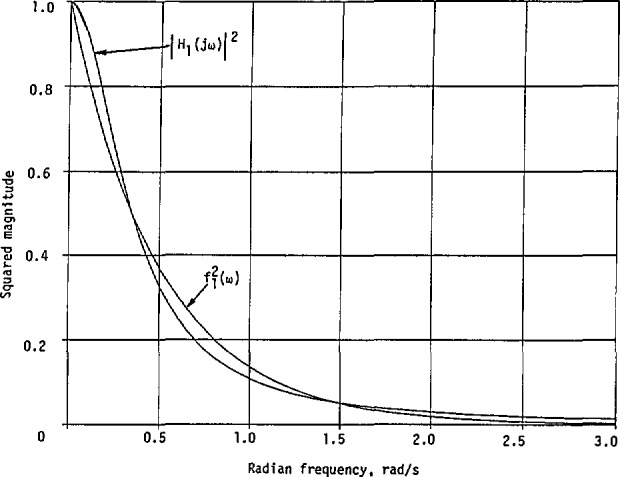
Comparison of functions given in Eqs. (91) and (93).

Since the maximum of $f_{1}^{2}(\omega )$ occurs at *ω* = 0 with $f_{1}^{2}(0)=1$, and the half-power frequency is *ω*_2_ = 0.3466, we approximate $f_{1}^{2}(\omega )$ by a linear system with lumped-constant elements represented by a squared-magnitude function in the form of Eq. (85),

$${\left|H_{1}(j\omega )\right|}^{2}=\frac{0.3466^{2}}{\omega^{2}+0.3466^{2}},$$ (93)

which is plotted in Fig. 5 together with $f_{1}^{2}(\omega )$ to show the approximation involved.

**Fig. 6:**
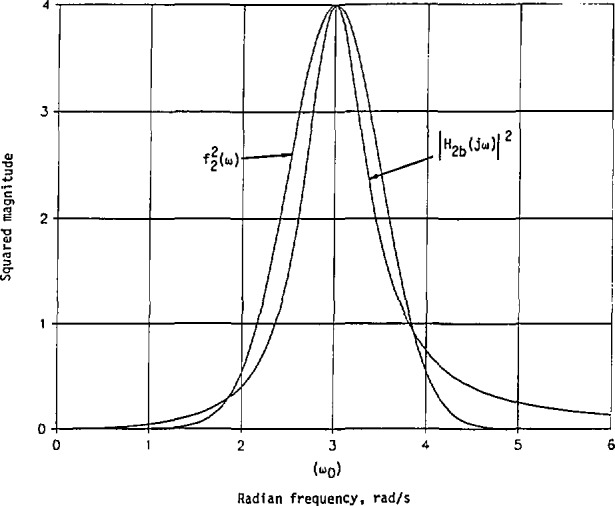
Comparison of functions given in Eqs. (92) and (94).

For $f_{2}^{2}(\omega )$, we have *ω*_0_ = 3 with $f_{2}^{2}(3)=4$. The half-power frequencies *ω*_1_ = 2.4113 and *ω*_2_ = 3.5887) have been obtained in example 2. Even though the relation $\omega_{0}^{2}=\omega_{1}\omega_{2}$ is not satisfied, this time we choose *c* = 0 in *H*_2_*_b_*(*s*) to make a better approximation at *ω* = 0. The other required parameters are determined from Eqs. (55) and (57): $b=\omega_{0}^{2}=9$, $a^{2}=\omega_{1}^{2}+\omega_{2}^{2}-2\omega_{0}^{2}=0.6931$, *a* = 0.8325, and $A^{2}=a^{2}f_{2}^{2}(3)=2.7725$. We then have the following to approximate the given $f_{2}^{2}(\omega )$:

$${\left|H_{2b}(j\omega )\right|}^{2}=\frac{2.7725\omega^{2}}{\omega^{4}-17.3069\omega^{2}+81},$$ (94)

which is plotted in Fig. 6 together with the given $f_{2}^{2}(\omega )$ for comparison purpose. The approximation shown here may be compared with that shown in Table 2, where *c* is chosen not equal to 0.

**Fig. 7:**
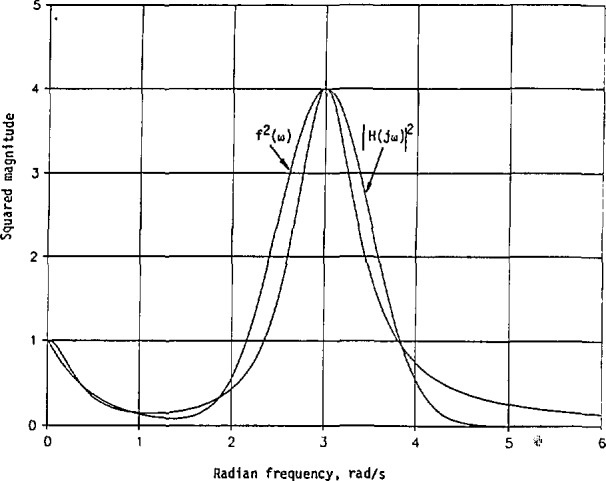
Comparison of the functions given in Eqs. (90) and (95).

The total squared magnitude to approximate *f*^2^(*ω*) in Eq. (90) is then given by the sum of expressions in Eqs. (93) and (94):

$$\begin{array}{l} {\left|H(j\omega )\right|}^{2}=\frac{0.1201}{\omega^{2}+0.1201}+\frac{2.7725\omega^{2}}{\omega^{4}-17.3069\omega^{2}+81}\\ \;=\frac{2.8926(\omega^{4}-0.6036\omega^{2}+3.3639)}{\left(\omega^{2}+0.1201)(\omega^{4}-17.3069\omega^{2}+81\right)}\\ \;=H(s)H(-s){|}_{s=j\omega }, \end{array}$$ (95)

which is plotted in Fig. 7 together with *f*^2^(*ω*).

From Eq. (95) we can apply the classical method in network theory [8] to extract the transfer functions as follows:

$$\begin{array}{l} H(s)H(-s)=\frac{2.8926(s^{4}+0.6036s^{2}+3.3639)}{\left(0.1201-s^{2})(s^{4}+17.3069s^{2}+81\right)}\\ \;=\frac{2.8926N(s)}{D(s)}, \end{array}$$ (96)

where

$$\begin{array}{l} N(s)=(s^{2}+1.7506s+1.8341)\\ \;(s^{2}-1.7506s+1.8341), \end{array}$$ (97)

and

$$\begin{array}{l} D(s)=(0.3466+s)(0.3466-s)\times \\ \;(s^{2}+0.8325s+9)(s^{2}-0.8325s+9). \end{array}$$ (98)

From the formats specifically expressed in Eqs. (97) and (98), we can assign appropriate factors to *H*(*s*) and *H*(−*s*). Obviously, the factors with positive signs in Eq. (98) have to be assigned to the denominator of *H*(*s*) because the poles are required to be in the left half of the s-plane for the linear system to be stable. The remaining factors in Eq. (98) with negative signs belong to the denominator of *H*(−*s*). However, either factor in Eq. (97) can be assigned to the numerator of *H*(*s*) because the zeros can be in the left-half or right-half plane. If the zeros are in the left half-plane, the system is at minimum phase. If they are in the right half-plane, the system is at nonminimum phase. Thus, we obtain the minimum-phase system,

$$H_{m}(s)=\frac{1.7008(s^{2}+1.7506s+1.8341)}{\left(s^{2}+0.3466)(s^{2}+0.8325s+9\right)}=1.7008[0.1526/(s+0.3466)+(0.8474s+1.3299)/(s^{2}+0.8325s+9)],$$ (99)

and the nonminimum-phase system,

$$H_{n}(s)=\frac{1.7008(s^{2}-1.7506s+1.8341)}{\left(s+0.3466)(s^{2}+0.8325s+9\right)}=1.7008[0.2900/(s+0.3466)+(0.7100s-2.2381)/(s^{2}+0.8325s+9)].$$ (100)

We can verify that |*H_m_*(*j*ω)|^2^ = |*H_n_*(*j*ω)|^2^ and that they both equal the squared magnitude in Eq. (95). Now, the nonminimum-phase transfer function in Eq. (100) can also be expressed in terms of the minimum-phase transfer function in Eq. (99) and an all-pass function

$$H_{n}(s)=H_{m}(s)H_{\text{all}}(s),$$ (101)

where

$$H_{\text{all}}(s)=(s^{2}-1.7506s+1.8341)/(s^{2}+1.7506s+1.8341),$$ (102)

is a second-order all-pass function with the complex-pair zeros in the right half-plane as mirror images of the poles in the left half-plane.

The associated phase functions are respectively

$$\theta_{m}(\omega )=\theta_{1}(\omega )+\theta_{2}(\omega )-\theta_{3}(\omega )$$ (103)

and

$$\theta_{n}(\omega )=\theta_{1}(\omega )+\theta_{2}(\omega )-\theta_{3}(\omega ),$$ (104)

where θ_1_ and θ_2_ are due to the denominator factors in Eq. (99) and *di* is due to the numerator in Eq. (99). They are:

$$\begin{array}{l} \theta_{1}(\omega )=\tan^{-1}(\omega /0.3466),\\ \theta_{2}(\omega )=\tan^{-1}[0.8325\omega /(9-\omega^{2})], \end{array}$$

and

$$\theta_{3}(\omega )=\tan^{-1}[1.7506\omega /(1.8341-\omega^{2})].$$ (105)

The minimum phase θ_m_(ω) in Eq. (103) can also be obtained from Eq. (95) with the Hilbert transform, as demonstrated before.

The impulse responses are:

$$h_{m}(t)/1.7008=0.1526e^{-0.3466t}+[0.8474\cos(2.9710t)+0.3289\sin(2.9710t)e^{-0.4163t}$$ (106)

and

$$h_{n}(t)/1.7008=0.2900e^{-0.3466t}+[0.7100\cos(2.9710t)-0.8528\sin(2.9710t)e^{-0.4163t}$$ (107)

where both *h*_m_(*t*) and *h*_n_(*t*) are normalized with respect to the common constant factor 1.7008.

**Fig. 8:**
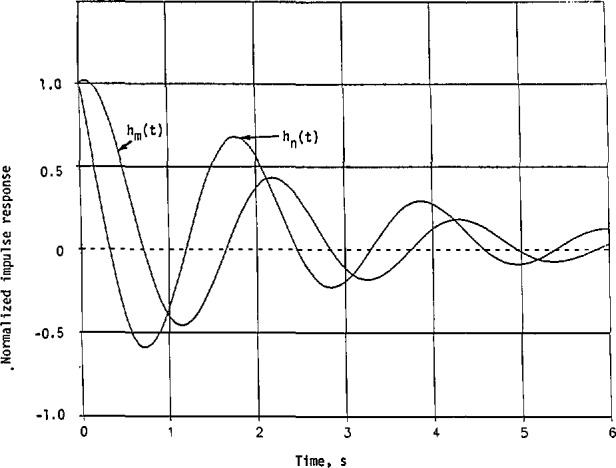
Normalized Impulse responses of the linear system with its given cw squared-magnitude response in Eq. (90) and the approximate squared magnitude in Eq. (95).

The normalized impulse responses in Eqs. (106) and (107) are plotted in Fig. 8. Here, they both start with 1 at *t = 0.* The normalized *h*_n_(*t*) decreases much faster than the normalized *h*_m_(*t*). Thus, *h*_n_(*t*) has a narrower beamwidth than *h*_m_(*t*), or less energy is concentrated initially with *h*_n_(*t*) than with *h*_m_(*t*).

The procedures demonstrated in example 3 can be easily extended to cases with more resonant frequencies, where there will be more terms in Eq. (89). More algebraic processes will be involved. When extracting transfer functions from the approximate squared magnitude, we will find more combinations for those with nonminimum phases while there is still only one solution for the transfer function with a minimum phase. Once the transfer functions are obtained, the remaining task for determining the corresponding phases and impulse responses is relatively straightforward. Determination of passive elements and a specific circuit structure to represent the extracted transfer function is a typical network synthesis problem [4, 8, 9], which is not within our scope of analysis. Once a network is synthesized, we can then use this model to make further analysis and even measurement of the network response due to any excitation, cw or pulse, with little effort and cost.

One caution, however, must be exercised for dealing with the cases where two resonant frequencies happen to be very close together. In these cases, the half-power frequencies associated with each resonant frequency must be entered into the computing process with smaller values than the actual values such that the final approximate squared magnitude still exhibits two distinct maxima at those resonant frequencies (rather than smeared together to have only one maximum).

The three examples presented so far are simulations where the given magnitudes are expressed in terms of neat mathematical functions and the resonant frequencies are small and easily manipulable numbers. In the real world, this is definitely not the case. Our goal is still to deduce an approximate squared magnitude from the given measured cw magnitude data so that a set of transfer functions and related characteristics can be determined and analyzed. Another example under this situation will be presented later in Sec. 5.

## 5. Frequency Transformation

In Sec. 4 the variable *ω* was loosely called frequency. Strictly speaking, *ω* is the normalized radian frequency. It can be translated into any frequency of interest by a simple frequency transformation [8],

$$\omega^{\prime }=B\omega ,$$ (108)

where *B* is a normalization constant, *ω* is the normalized radian frequency, and *ω′* is the actual radian frequency.

In presenting example 1 in Sec. 4.1, we cited *ω*_0_ = 2 as the resonant frequency. If the actual resonant frequency occurs at 20 MHz, we should have used *ω*_0_ = 4*π*(10)^7^ rad. Instead, we chose then to use *ω*_0_ = 2 to avoid manipulations with large numbers. After obtaining |*H*_2_*_a_* (*jω*)|^2^, *H*_2_*_a_*(*s*), θ_m_(*ω*), and *h*_2_*_a_*(*t*) in Eqs. (41) through (44), we can apply Eq. (108) with *B* = 2*π*(10)^7^ to transform the results from *ω* to *ω*′ with *ω = ω′*/*B.* Thus, the solutions in Eqs. (41–44) become respectively

$$\begin{array}{l} {\left|G_{2a}\left(j\omega^{\prime }\right)\right|}^{2}=\frac{0.6931B^{4}}{{\omega^{\prime }}^{4}-8B^{2}{\omega^{\prime }}^{2}+16.6931B^{4}}\\ \;=G_{2a}\left(s^{\prime }\right)G_{2a}\left(-s^{\prime }\right){|}_{s^{\prime }=j\omega^{\prime }}, \end{array}$$

$$\begin{array}{l} G_{2a}\left(s^{\prime }\right)=\frac{0.8326B^{2}}{{s^{\prime }}^{2}+0.4141Bs^{\prime }+4.0857B^{2}},\\ \theta_{m}\left(\omega^{\prime }\right)=\tan^{-1}\left[0.4141B\omega^{\prime }/\left(4.0857B^{2}-{\omega^{\prime }}^{2}\right)\right], \end{array}$$ (109)

and

$$h_{2a}\left(t\right)=0.4141B\;e^{-0.2070Bt}\sin\left(1.0107Bt\right).$$

**Fig. 9:**
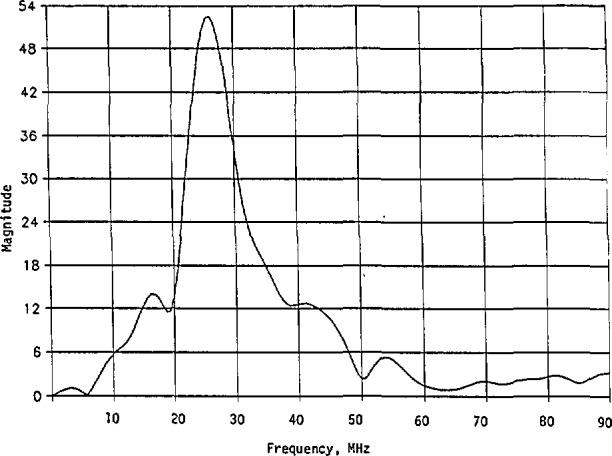
Measured electric-field magnitude (vertical polarization) reflected from a helicopter when radiated by an external impulse signal.

These procedures apply also to the other type of second-order and higher-order transfer functions. With this explained, we now are ready to give another example based on the real-world data shown in Fig. 9. The data represent the measured but normalized electric fields (magnitude) of vertical polarization, reflected from a helicopter when it is irradiated by an impulse signal. By examining the curve in Fig. 9, we notice four significant resonant frequencies at 16.50, 26.25, 41.00, and 53.375 MHz. The frequency near 3 MHz is ignored because its magnitude response is rather small (close to background noise). It can, however, be added if necessary.

To simplify the analysis, we temporarily designate *ω*_01_ = 2*π* × 16.50 = 103.6726, *ω*_02_ = 52.5*π* = 164.9336, *ω*_03_ = 82*π* = 257.6106, and *ω*_04_ = 106.75*π* = 335.3650. From Fig. 9, we also see that the respective maximum responses at these resonant frequencies are 14.00 (22.92 dB), 52.52 (34.41 dB), 13.00 (22.28 dB), and 5.33 (14.53 dB). Their half-power frequencies are approximately *ω*_11_ = 29*π* = 91.1062, *ω*_12_ = 36*π* = 113.0973; *ω*_21_ = 46*π* = 144.5132, *ω*_22_ = 59*π* = 185.3540; *ω*_31_ = 76*π* = 238.7610, *ω*_32_ = 90*π* = 282.7433; and *ω*_41_ = 104*π* = 326.7256, *ω*_42_ = 108.6*π* = 341.1770. Here the first subscript refers to the resonant frequency, the second subscript 1 refers to the half-power frequency on the left side of the respective resonant frequency, and the second subscript 2 refers to the half-power frequency on the right side of the respective resonant frequency. The two half-power frequencies, *ω*_12_ = 36*π* and *ω*_31_ = 76*π*, are not real, but extrapolated for the analysis. Also, we later use *B* = 10^6^, in accordance with Eq. (108), as the transformation constant.

Even though the condition $\omega_{1}^{2}+\omega_{2}^{2}=2\omega_{0}^{2}$ is not exactly satisfied at either of the four resonant frequencies, we choose to use the type of second-order transfer function in Eq. (10a) for obtaining the approximate squared magnitudes. Using Eq. (23), we have for the first resonant frequency at *ω*_01_,

$$A_{1}=3.1435\left(10^{4}\right),b_{1}=1.0980\left(10^{4}\right),a_{1}=21.5420,$$

and

$${\left|H_{1}\left(j\omega \right)\right|}^{2}=9.8816\left(10^{8}\right)/\left[\omega^{4}-2.1496\left(10^{4}\right)\omega^{2}+1.2056\left(10^{8}\right)\right];$$ (110)

for the second resonant frequency at *ω*_02_,

$$A_{2}=3.5377\left(10^{5}\right),b_{2}=2.8025\left(10^{4}\right),a_{2}=40.5358,$$

and

$${\left|H_{2}\left(j\omega \right)\right|}^{2}=12.5153\left(10^{10}\right)/\left[\omega^{4}-5.4406\left(10^{4}\right)\omega^{2}+7.8538\left(10^{8}\right)\right];$$ (111)

for the third resonant frequency at *ω*_03_,

$$A_{3}=1.4909\left(10^{5}\right),b_{3}=6.7347\left(10^{4}\right),a_{3}=44.3546,$$

and

$${\left|H_{3}\left(j\omega \right)\right|}^{2}=2.2228\left(10^{10}\right)/\left[\omega^{4}-1.3273\left(10^{5}\right)\omega^{2}+4.5356\left(10^{9}\right)\right];$$ (112)

and for the last resonant frequency at *ω*_04_,

$$A_{4}=2.5723\left(10^{4}\right),b_{4}=1.1257\left(10^{5}\right),a_{4}=14.3871,$$

and

$${\left|H_{4}\left(j\omega \right)\right|}^{2}=6.6166\left(10^{8}\right)/\left[\omega^{4}-2.2494\left(10^{5}\right)\omega^{2}+1.2673\left(10^{10}\right)\right];$$ (113)

**Fig. 10:**
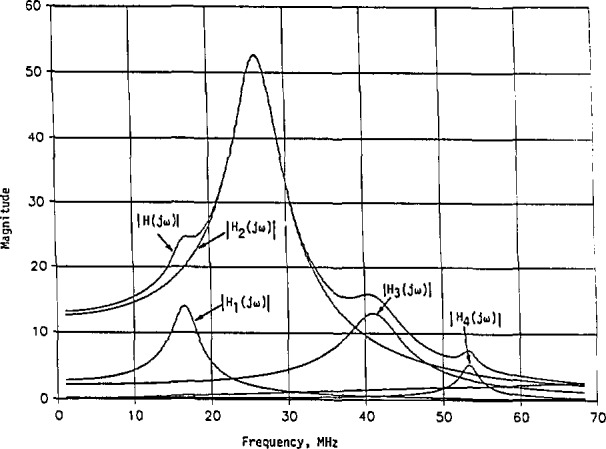
The approximate magnitude to that in Fig. 9, together with component magnitude functions identified for each resonant frequency, where |*H*(*jω*)|=[|*H*_1_(*jω*)|^2^+|*H*_2_(*jω*)|^2^+|*H*_3_(*jω*)|^2^+|*H*_4_(*jω*)|^2^]^1/2^, Eq. (114).

The final approximate squared magnitude is then

$$\begin{array}{l} {\left|H\left(j\omega \right)\right|}^{2}={\left|H_{1}\left(j\omega \right)\right|}^{2}+{\left|H_{2}\left(j\omega \right)\right|}^{2}+{\left|H_{3}\left(j\omega \right)\right|}^{2}+{\left|H_{4}\left(j\omega \right)\right|}^{2}\\ \;=1.4903\left(10^{11}\right)N\left(\omega^{2}\right)/D\left(\omega^{2}\right), \end{array}$$ (114)

where

$$\begin{array}{l} N\left(\omega^{2}\right)=\omega^{12}-3.6694\left(10^{5}\right)\omega^{10}+5.1348\left(10^{10}\right)\omega^{8}\\ \;-3.4113\left(10^{15}\right)\omega^{6}+1.0820\left(10^{20}\right)\omega^{4}\\ \;-1.3940\left(10^{24}\right)\omega^{2}+6.2996\left(10^{27}\right)\\ \;=\left[\omega^{4}-2.1747\left(10^{4}\right)\omega^{2}+1.2577\left(10^{8}\right)\right]\\ \;\times \left[\omega^{4}-1.2098\left(10^{5}\right)\omega^{2}+3.9741\left(10^{9}\right)\right]\\ \;\times \left[\omega^{4}-2.242\left(10^{5}\right)\omega^{2}+1.2615\left(10^{10}\right)\right], \end{array}$$ (115)

and D(*ω*^2^) is the product of the four denominators in Eqs. (110) through (113). The magnitude in Eq. (114) is shown in Fig. 10 together with the component magnitude functions obtained in Eqs. (110) through (113). Comparing Figs. 9 and 10, we see, except the frequency scale, the approximation in Eq. (114) is generally very good. The dominant features at *ω*_02_ and its half-power frequencies are indeed excellent. The shifts in *ω*_01_, and *ω*_03_, are minor. The position of *ω*_04_ remains practically the same. The only major changes are the magnitudes at *ω* = 0 and *ω*_01_. This deficiency can be improved if we choose the second-order transfer function of Eq. (10b) or the approximate squared-magnitude function in Eq. (49) with *c* = 0 at the beginning for |*H_2_*(*jω*)|^2^. From Eq. (115) we already see the large coefficients even when we used the normalized frequency to begin with. If we wish to convert the frequency into megahertz, the numerator in Eq. (114) will become

$$\begin{array}{l} \left(1/B^{12}\right)\left[\omega^{4}-2.1747\left(10^{4}\right)B^{2}\omega^{2}+1.2577\left(10^{8}\right)B^{4}\right]\\ \;\times \left[\omega^{4}-1.2098\left(10^{5}\right)B^{2}\omega^{2}+3.9741\left(10^{9}\right)B^{4}\right]\\ \;\times \left[\omega^{4}-2.2422\left(10^{5}\right)B^{2}\omega^{2}+1.2615\left(10^{10}\right)B^{4}\right], \end{array}$$ (116)

and the denominator of Eq. (114) will become

$$\begin{array}{c} \left(1/B^{16})[\omega^{4}-2.1496(10^{4})B^{2}\omega^{2}+1.2056(10^{8})B^{4}\right]\\ \;\times \;[\omega^{4}-5.4406(10^{4})B^{2}\omega^{2}+7.8538(10^{8})B^{4}]\\ \;\times \;[\omega^{4}-1.3272(10^{5})B^{2}\omega^{2}+4.5356(10^{9})B^{4}]\\ \;\times \;[\omega^{4}-2.2494(10^{5})B^{2}\omega^{2}+1.2673(10^{10})B^{4}], \end{array}$$ (117)

where *B* =10^6^.

Referring to Eq. (114) and setting |*H*(*jω*)|^2^ =*H(s)H*(−*s*)|*_s_*_=_*_jω_*, we obtain

$$H(s)H(-s)=1.4903(10^{11})N(-s^{2})/D(-s^{2}),$$ (118)

where

$$N(-s^{2})=N_{1}(+)N_{2}(+)N_{3}(+)N_{1}(-)N_{2}(-)N_{3}(-),$$ (119)

and

$$D(-s^{2})=D_{1}(+)D_{2}(+)D_{3}(+)D_{4}(+)D_{1}(-)\times D_{2}(-)D_{3}(-)D_{4}(-),$$ (120)

with

$$\begin{array}{l} N_{1}(+)=s^{2}+26.1252s+1.1215(10^{4}),\\ N_{1}(-)=s^{2}-26.1252s+1.1215(10^{4}), \end{array}$$

$$\begin{array}{l} N_{2}(+)=s^{2}+71.4229s+6.3040(10^{4}),\\ N_{2}(-)=s^{2}-71.4229s+6.3040(10^{4}); \end{array}$$

$$\begin{array}{l} N_{3}(+)=s^{2}+20.5139s+1.1232(10^{5}),\\ N_{3}(-)=s^{2}-20.5139s+1.1232(10^{5}); \end{array}$$

$$\begin{array}{l} D_{1}(+)=s^{2}+21.5420s+1.0980(10^{4}),\\ D_{1}(-)=s^{2}-21.5420s+1.0980(10^{4}), \end{array}$$

$$\begin{array}{l} D_{2}(+)=s^{2}+40.5358s+2.8025(10^{4}),\\ D_{2}(-)=s^{2}-40.5358s+2.8025(10^{4}), \end{array}$$

$$\begin{array}{l} D_{3}(+)=s^{2}+44.3546s+6.7347(10^{4}),\\ D_{3}(-)=s^{2}-44.3546s+6.7347(10^{4}), \end{array}$$

$$\begin{array}{l} D_{4}(+)=s^{2}+14.3871s+1.1257(10^{5}),\\ D_{4}(-)=s^{2}-14.3871s+1.1257(10^{5}). \end{array}$$

Since we require the system to be stable (no poles in the right half of the *s*-plane), we have to assign *D*_1_(+)*D*_2_(+)*D*_3_(+)*D*_4_(+) as the denominator for *H(s).* Thus, *D*_1_(−)*D*_2_(−)*D*_3_(−)*D*_4_(−) belongs to *H*(−*s*). As far as the numerator for *H(s)* is concerned, we have many choices from Eq. (119). When *N*_1_(+)*N*_2_(+)*N*_3_(+) is assigned as the numerator of *H*(*s*), *N*_1_(−)*N*_2_(−)*N*_3_(−) then belongs to *H*(−*s*). In this case, there are no zeros in the right half of the *s*-plane. The result is a minimum-phase transfer function. We then have

$$\begin{array}{c} H_{m}(s)=3.8604(10^{5})N_{1}(+)N_{2}(+)N_{3}(+)/[D_{1}(+)D_{2}(+)D_{3}(+)D_{4}(+)]\\ =3.8604(10^{5})\left\{\frac{F_{1}s+G_{1}}{D_{1}(+)}+\frac{F_{2}s+G_{2}}{D_{2}(+)}+\frac{F_{3}s+G_{3}}{D_{3}(+)}+\frac{F_{4}s+G_{4}}{D_{4}(+)}\right\}, \end{array}$$ (121)

where

$$F_{1}=2.3245/10^{4},\;G_{1}=1.3664/10^{2},$$

$$F_{2}=5.3398/10^{4},\;G_{2}=0.8742,$$

$$F_{3}=-6.8335/10^{4},\;G_{3}=0.1055,$$

$$F_{4}=-8.3091/10^{5},\;G_{4}=1.7752/10^{3}.$$

Applying the following two Laplace transform pairs [9] to Eq. (121):

$$(s+\alpha )/[{\left(s+\alpha \right)}^{2}+\beta^{2}]↔e^{-\alpha t}\cos\beta t,$$ (122a)

and

$$1/[{\left(s+\alpha \right)}^{2}+\beta^{2}]↔(1/\beta )e^{-\alpha t}\sin\beta t,$$ (122b)

we obtain the impulse response of this system,

$$\begin{array}{l} h_{m}(t)=3.8604(10^{2})\{\left(0.2325\cos\beta_{1}t+0.1071\sin\beta_{1}t\right)\\ \;\times e^{-\alpha_{1}t}+(0.5340\cos\beta_{2}t+5.1959\sin\beta_{2}t)e^{-\alpha_{2}t}\\ \;+(-0.6833\cos\beta_{3}t+0.4665\sin\beta_{3}t)e^{-\alpha_{3}t}\\ \;+(-0.0831\cos\beta_{4}t+0.0071\sin\beta_{4}t)e^{-\alpha_{4}t}\}, \end{array}$$ (123)

where

$$\alpha_{i}=a_{i}/2,\;\text{and}\;\beta_{i}=\sqrt{b_{i}-{\left(a_{i}/2\right)}^{2}},i=1,2,3,\;\text{and}\;4.$$

More specifically, we have

$$\begin{array}{lll} \alpha_{1}=10.7710, & \alpha_{2}=20.2679, & \alpha_{3}=22.1773,\\ \alpha_{4}=7.1936, & \beta_{1}=104.2305, & \beta_{2}=166.1740,\\ \beta_{3}=258.5634, & \beta_{4}=335.4422. & \end{array}$$

Equation (123) shows that at *t* =0, *h*_m_(*t*) = 0. This agrees with the result predicted by the initial-value theorem [10, 12]. The largest coefficient is with sin *β*_2_*t* associated with the second resonant frequency. This is obvious when we refer to Fig. 10 where *ω*_02_ is dominant.

**Fig. 11:**
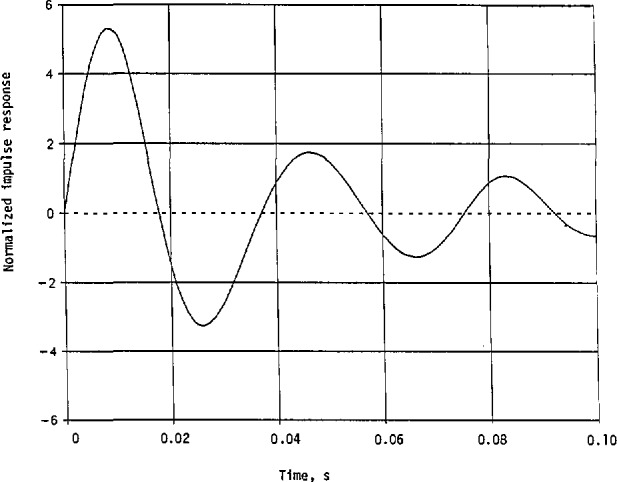
Normalized impulse response of the linear system whose approximate magnitude of the transfer function is shown in Fig. 10. This is for the minimum-phase case with its transfer function given in Eq. (121).

With the impulse response so determined, the system’s response to a general excitation can then be computed by convolution integral [12]. When referring to frequencies in megahertz, we simply modify the impulse response in Eq. (123) by multiplying the coefficient 3.8604(10^2^), *α_i_*, and *β_i_*(*i* = 1,2,3,4) by the transformation constant *B* = 10^6^. The impulse response *h*_m_(*t*) before applying the frequency transformation is presented in Fig. 11, where the coefficient of 3.8604(10^2^) in Eq. (123) has been dropped. We see, from Fig. 11, that the period is about 0.019 *s*, giving 0.019 *β*_2_≈*π*. The major maximum occurs approximately at *t_i_* = 0.008 *s* with *h*_m_(*t*)/3.8604(10^2^) = 5.3094, and the second maximum occurs approximately at *t*_2_ = 0.026 *s* with *h*_m_(*t*_2_)/3.8604(100= −3.2652. The ratio *of h*_m_(*t*_1_)*/*|*h*_m_(*t*_2_)| = 1.6261, which is close to $e^{\alpha_{2}(t_{2}-t_{1})}$. Thus, the second resonant frequency is, indeed, the dominant one [14].

The associated phase is given by

$$\theta_{m}(\omega )=\theta_{1}(\omega )+\theta_{2}(\omega )+\theta_{3}(\omega )+\theta_{4}(\omega )-\theta_{5}(\omega )-\theta_{6}(\omega )-\theta_{7}(\omega ),$$ (124)

where the first four component phases are due to *D*_1_(+)*D*_2_(+)*D*_3_(+)*D*_4_(+), and the last three are due to *N*_1_(+)*N*_2_(+)*N*_3_(+). That is,

$$\begin{array}{l} \theta_{1}(\omega )=\tan^{-1}\left[\frac{21.5420\omega }{1.0980(10^{4})-\omega^{2}}\right],\\ \theta_{2}(\omega )=\tan^{-1}\left[\frac{40.5358\omega }{2.8025(10^{4})-\omega^{2}}\right],\\ \theta_{3}(\omega )=\tan^{-1}\left[\frac{44.3546\omega }{6.7347(10^{4})-\omega^{2}}\right],\\ \theta_{4}(\omega )=\tan^{-1}\left[\frac{14.3871\omega }{1.1257(10^{5})-\omega^{2}}\right],\\ \theta_{5}(\omega )=\tan^{-1}\left[\frac{26.1252\omega }{1.1215(10^{4})-\omega^{2}}\right],\\ \theta_{6}(\omega )=\tan^{-1}\left[\frac{71.4229\omega }{6.3040(10^{4})-\omega^{2}}\right], \end{array}$$

and

$$\theta_{7}(\omega )=\tan^{-1}\left[\frac{20.5139\omega }{1.1232(10^{5})-\omega^{2}}\right].$$ (125)

**Fig. 12:**
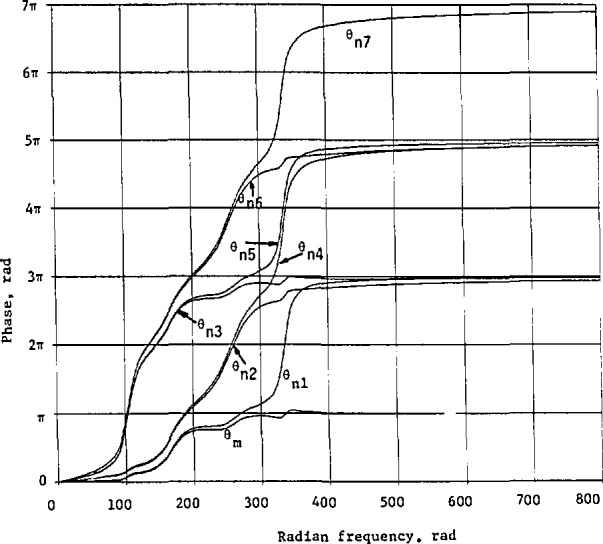
Phases of the sample linear system whose approximate cw magnitude response is shown in Fig. 10.

Expressing the phase in terms of megahertz, we multiply the numerator inside the arctangents by the normalization constant *B* and the constant term in the denominator by *B*^2^. The minimum phase *θ*_m_ in Eq. (124) before frequency transformation is presented in Fig. 12.

Seven other possible solutions for the transfer function with nonminimum phases can be obtained from Eqs. (118) and (119) as

$$\begin{array}{l} H_{n1}(s)=CN_{1}(+)N_{2}(+)N_{3}(-),\\ H_{n2}(s)=CN_{1}(+)N_{2}(-)N_{3}(+),\\ H_{n3}(s)=CN_{1}(-)N_{2}(+)N_{3}(+),\\ H_{n4}(s)=CN_{1}(+)N_{2}(-)N_{3}(-),\\ H_{n5}(s)=CN_{1}(-)N_{2}(+)N_{3}(-),\\ H_{n6}(s)=CN_{1}(-)N_{2}(-)N_{3}(+), \end{array}$$

and

$$H_{n7}(s)=CN_{1}(-)N_{2}(-)N_{3}(-),$$ (126)

where

$$C=3.8604(10^{5})/[D_{1}(+)D_{2}(+)D_{3}(+)D_{4}(+)].$$

Following the same procedures of partial fractions as in Eq. (121), we list the expansion coefficients for the 7 nonminimum-phase cases in Table 3.

The corresponding impulse responses also take the same form as in Eq. (123) with the same *α_i_*,- and *β_i_* but with different coefficients associated with the cosine and sine terms. These coefficients are listed in Table 4.

These impulse responses with nonminimum phases, without including the frequency transformation, are shown in Figs. 13 (a) and (b) to compare with that in Fig. 11 for the minimum-phase case. It happens that the first maxima *of h*_ni_(*t*) are all below the first maximum *of h*_m_(*t*), and that the first nulls *of h*_ni_(*t*) are also closer to the origin than the first null *of h*_m_(*t*). More energy is concentrated near *t=0* in *h*_m_(*t*) than any of the *h*_ni_(*t*), *i* = 1,2,…, 7.

The phases associated with the transfer function in Eq. (126) are respectively:

$$\begin{array}{l} \theta_{n1}=\theta_{1}+\theta_{2}+\theta_{3}+\theta_{4}-\theta_{5}-\theta_{6}+\theta_{7},\\ \theta_{n2}=\theta_{1}+\theta_{2}+\theta_{3}+\theta_{4}-\theta_{5}+\theta_{6}-\theta_{7},\\ \theta_{n3}=\theta_{1}+\theta_{2}+\theta_{3}+\theta_{4}+\theta_{5}-\theta_{6}-\theta_{7},\\ \theta_{n4}=\theta_{1}+\theta_{2}+\theta_{3}+\theta_{4}-\theta_{5}+\theta_{6}+\theta_{7},\\ \theta_{n5}=\theta_{1}+\theta_{2}+\theta_{3}+\theta_{4}+\theta_{5}-\theta_{6}+\theta_{7},\\ \theta_{n6}=\theta_{1}+\theta_{2}+\theta_{3}+\theta_{4}+\theta_{5}+\theta_{6}-\theta_{7}, \end{array}$$

and

$$\theta_{n7}=\theta_{1}+\theta_{2}+\theta_{3}+\theta_{4}+\theta_{5}+\theta_{6}+\theta_{7},$$ (127)

where

*θ_i_*, *i* =1,2,…, 7, are given in Eq. (125) before normalization.

Graphs for *θ*_ni_ are also plotted in Fig. 12 for comparison purpose. Clearly we see that *θ*_ni_ > *θ*_m_, because each component phase given in Eq. (125) is nonnegative, varying from 0 to *π* as *ω* varies from 0 to ∞.

## 6. Consideration of Energy Contents

To assess the ability of a system to withstand damage from an external unwanted excitation, it is often useful to compute the energy content associated with an impulse response [15]. Indeed, *if h*(*t*) represents a voltage waveform across a 1 Ω resistor, the quantity

$$E=\int_{0}^{\infty }h^{2}(t)dt,$$ (128)

equals the total energy delivered to the resistor by the impulsive excitation [2]. Equation (128) also represents the area under the curve *h*^2^(*t*).

The energy *E* may also be computed, in view of Parseval’s theorem [2], by

$$E=\frac{1}{2\pi }\int_{-\infty }^{\infty }{\left|H(j\omega )\right|}^{2}d\omega .$$ (129)

Thus, when the minimum-phase impulse response *h*_m_(*t*) and the associated nonminimum-phase impulse response *h*_n_(*t*) have an identical |*H*(*jω*)|^2^, their respective total energies [in 0⩽*t*⩽∞] are equal even though *h*_n_(*t*) < *h*_m_(*t*) during the initial period near *t* = 0 +, as discussed in Sec. 4. These facts can also be demonstrated by referring to the examples given earlier.

For example 1 in Eq. (40), |*H*_2_*_a_*(*jω*)|^2^ is given in Eq. (41). Its energy content, according to Eq. (129), is

$$\begin{array}{l} E=\frac{0.6931}{2\pi }\int_{-\infty }^{\infty }\frac{d\omega }{\omega^{4}-8\omega^{2}+16.6931}\\ \;=\frac{0.6931}{\pi }\int_{-\infty }^{\infty }\frac{d\omega }{\left(\omega^{2}+p^{2}\right)+\left(\omega^{2}+p{*}^{2}\right)}\\ \;=\frac{0.6931}{j\;1.6650\pi }\int_{-\infty }^{\infty }\left[\frac{1}{\omega^{2}+p^{2}}-\frac{1}{\omega^{2}+p{*}^{2}}\right]\;d\omega \\ \;=0.2049, \end{array}$$ (130)

where

$$p^{2}=-4-j\;0.8325,\;\text{and}$$

p*=complex conjugate ofp.

The last step in Eq. (130) is obtained by using

$$\begin{array}{l} \int_{0}^{\infty }\frac{\cos(qx)dx}{x^{2}+p^{2}}=\frac{\pi }{2p}e^{-qp},\\ \text{with}\;q\;⩾\;0\\ \text{and}\;Re(p)>0. \end{array}$$ (131)

We can also obtain the energy by referring to the impulse response given in Eq. (44) for the minimum-phase case,

$$\begin{array}{l} E_{m}=\int_{0}^{\infty }h_{2a}^{2}(t)dt\\ \;=0.1715\int_{0}^{\infty }e^{-0.4140t}\sin^{2}(2.0107t)dt\\ \;=0.2049, \end{array}$$ (132)

which is indeed the same as in Eq. (130).

Using the corresponding impulse response given in Eq. (47) for the nonminimum-phase case yields the same result. That is,

$$E_{n}=\int_{0}^{\infty }h_{n}^{2}(t)dt=0.2049.$$ (133)

For example 2 presented in Eq. (75), the corresponding |*H*_2_*_b_* (*jω*)|^2^, *h*_m_(*t*), and *h*_n_(*t*) can be found respectively in Eqs. (76), (81), and (82). The total energy for this system is

$$\begin{array}{l} E=\frac{1}{2\pi }\int_{-\infty }^{\infty }{\left|H_{2b}(j\omega )\right|}^{2}d\omega \\ \;=\int_{0}^{\infty }h_{m}^{2}(t)\;dt\\ \;=\int_{0}^{\infty }h_{n}^{2}(t)\;dt\\ \;=2.3124. \end{array}$$ (134)

If we replace the upper integration limit ∞ in Eq. (128) by a finite *T*, we can analyze the energy content absorbed by the system during the initial period after an external excitation is applied. Referring again to example 1 with *h*_2_*_a_*(*t*) given in Eq. (44) and *h*_n_(*t*) in Eq. (47), and carrying out the details, we have

$$\begin{array}{l} h_{2a}^{2}(t)=0.1714\;e^{-\alpha_{1}t}\sin^{2}\beta t\\ \;=0.0857\;e^{-\alpha_{1}t}(1-\cos2\beta t), \end{array}$$

$$\begin{array}{l} h_{n}^{2}(t)=0.1270\;e^{-2t}-(0.2540\;\cos\beta t\\ \;+0.1950\;\sin\beta t)e^{-1.2070t}\\ \;+(0.1009+0.0261\;\cos2\beta t\\ \;+0.0975\;\sin2\beta t)e^{-\alpha_{1}t}, \end{array}$$

$$\begin{array}{c} E_{m}=\int_{0}^{T}h_{2a}^{2}(t)dt=0.2048-0.2070\;e^{-\alpha_{1}T}\\ -0.0052\;e^{-\alpha_{1}T}(-\alpha_{1}\cos2\beta T\\ +2\beta \sin2\beta T), \end{array}$$

and

$$\begin{array}{l} E_{n}=\int_{0}^{T}h_{n}^{2}(t)dt=0.1413+0.0635(1-e^{-2T})\\ \;+(-0.2437-0.0246\;\cos\;2\beta T\\ \;+0.0039\;\sin\;2\beta T)\;e^{-\alpha_{1}T}\\ \;+(0.1270\;\cos\;\beta T\\ \;-0.0501\;\sin\;\beta T)e^{-1.2070T}, \end{array}$$ (135)

where

$$\alpha_{1}=0.4141,\;and\;\beta =2.0107.$$

**Fig. 14:**
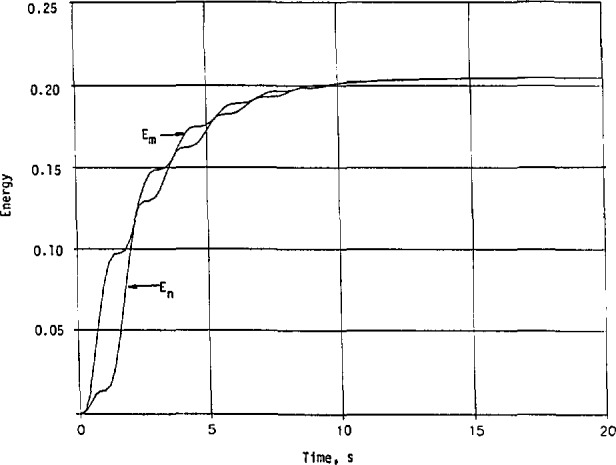
Energy contents of the sample system whose transfer functions are given in Eqs. (42) and (45).

Numerical results for both *E*_m_ and *E*_n_ are shown in Fig. 14 and indicate clearly that *E*_n_ < *E*_m_ [12]. They are equal only when *T* → ∞. This reconfirms that the impulse response and transfer function with a minimum phase deduced from a given magnitude can be used as the worst case for analysis purpose, as far as the initial impact to the system under study by an external unwanted source is concerned.

**Fig. 15:**
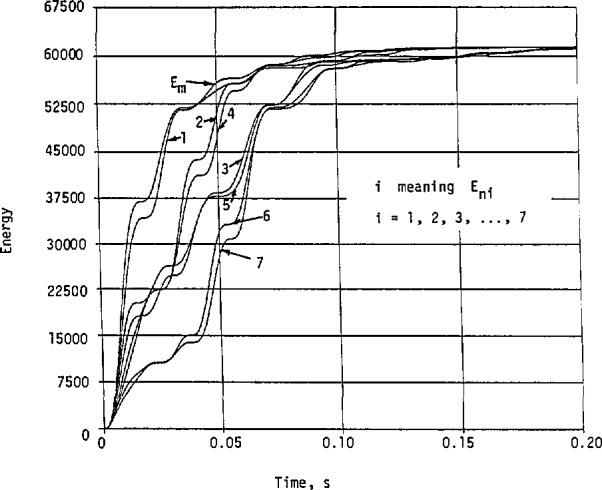
Energy contents of the helicopter whose approximate cw magnitude response is given in Fig. 10.

For the practical example shown in Figs. 9 and 10, we present the results on energy content in Fig. 15. Again, we have *E*_ni_ < *E*_m_, *i* = 1,2,…,7.

## 7. Conclusions

We have used a simple method known in classical network theory to determine the complete characteristics for an unknown linear system from a given cw magnitude response only. These characteristics include possible different transfer functions, their phases, and the corresponding impulse responses. Only one transfer function is minimum phase. The main achievement is to deduce an approximate squared-magnitude function in the form of a ratio of two even polynomials based on the outstanding features in the given magnitude response, such as resonant frequencies and bandwidths. The remaining procedures for obtaining the complete system characteristics are exact. Four examples, three simulations and one using measured data, have been given to illustrate the proposed method. We have written software that greatly facilitates application of this technique. It first performs numerical calculations necessary to obtain the system transfer functions from the measured magnitude-frequency input data, and then gives impulse responses. We also have shown that the minimum-phase case, through its associated impulse response and energy content, constitutes the most pessimistic estimate as far as the initial threat to the system is concerned.

## Figures and Tables

**Fig. 13 (a):**
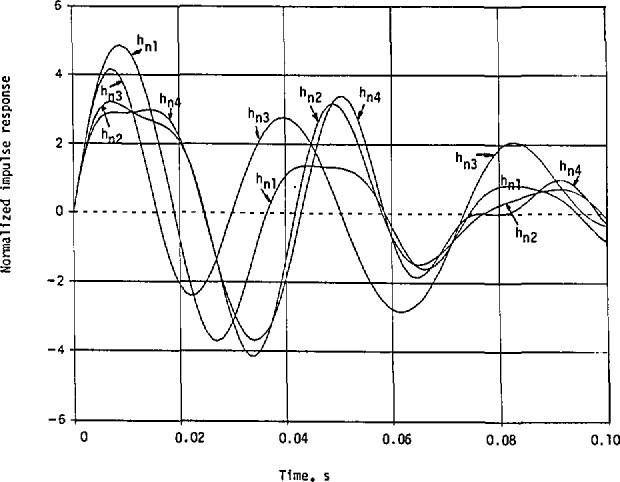
Normalized impulse response of the helicopter with the approximate magnitude response given in Fig. 10, but with the nonminimum-phase transfer functions given in Eq. (126).

**Fig. 13 (b):**
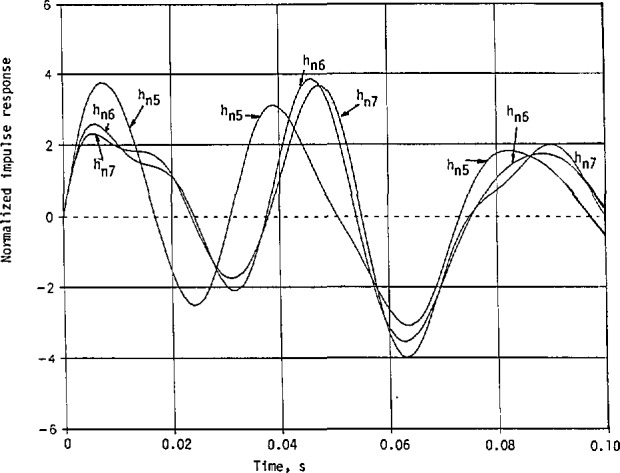
Normalized impulse response of the helicopter with the approximate magnitude resfwnse given in Fig. 10, but with the nonminimum-phase transfer functions given in Eq. (126). Continuation of Fig. 13(a).

**Table 1:** Approximation of the given function in Eq. (40) by the squared-magnitude function in Eq. (41)

*ω*	*f*^2^(*ω*)	|*H*_2_*_a_*(*jω*)|^2^	Error
0.00	0.0000	0.0415	0.0415
0.50	0.0000	0.0470	0.0470
1.00	0.0001	0.0715	0.0714
1.50	0.0468	0.1846	0.1378
1.75	0.4152	0.4409	0.0257
1.80	0.5612	0.5454	−0.0158
1.85	0.7164	0.6752	−0.0413
1.90	0.8589	0.8200	−0.0389
1.95	0.9617	0.9467	−0.0150
2.00	1.0000	1.0000	0.0000
2.05	0.9598	0.9441	−0.0157
2.10	0.8453	0.8048	−0.0405
2.15	0.6787	0.6414	−0.0373
2.20	0.4938	0.4955	0.0017
2.25	0.3234	0.3804	0.0570
2.50	0.0063	0.1204	0.1141
3.00	0.0000	0.0270	0.0270

**Table 2:** Approximation of the given function in Eq. (75) by the squared-magnitude function in Eq. (76)

*ω*	*f*^2^(*ω*)	|*H*_2_*_b_*(*jω*)|^2^	Error
0.00	0.0000	0.2809	0.2809
0.25	0.0000	0.2864	0.2864
0.50	0.0000	0.3037	0.3037
0.75	0.0002	0.3350	0.3348
1.00	0.0013	0.3848	0.3835
1.25	0.0087	0.4607	0.4520
1.50	0.0444	0.5768	0.5324
1.75	0.1757	0.7579	0.5822
2.00	0.5413	1.0493	0.5080
2.25	1.2986	1.5323	0.2337
2.50	2.4261	2.3206	−0.1055
2.75	3.5300	3.3844	−0.1456
3.00	4.0000	4.0000	0.0000
3.25	3.5300	3.3851	−0.1449
3.50	2.4261	2.3215	−0.1046
3.75	1.2986	1.5294	0.2308
4.00	0.5413	1.0396	0.4983
4.25	0.1757	0.7398	0.5641
4.50	0.0444	0.5495	0.5051
4.75	0.0087	0.4231	0.4144
5.00	0.0013	0.3356	0.3343
5.25	0.0002	0.2728	0.2726
5.50	0.0000	0.2264	0.2264

**Table 3:** Partial fraction expansion coefficients for nonminimum-phase transfer functions

Non-Min.	*F*_1_×10^3^	*G*_1_×10	*F*_2_×10^3^	*G*_2_	*F*_3_×10^3^	*G*_3_×10	*F*_4_×10^4^	*G*_4_×10
n1	0.2290	0.1470	0.1253	0.8842	−0.8235	0.6849	4.6925	0.1281
n2	0.2017	0.2034	−3.0480	0.8467	2.8954	1.1708	−0.4910	0.2434
n3	−2.5895	−0.0188	3.2415	0.8940	−0.5711	1.5046	−0.8103	0.0653
n4	0.1952	0.2124	−3.5248	0.8083	2.9958	2.9139	3.3377	−1.2197
n5	−2.6114	−0.1339	2.8849	0.9406	−0.7427	1.2150	4.6927	−0.1422
n6	−2.6634	−0.8067	−0.2450	1.1784	2.9444	−0.6197	−0.3600	0.2700
n7	−2.6541	−0.9251	−0.8178	1.1789	3.2050	1.0964	2.6693	−1.4034

**Table 4:** Coefficients associated with cosine and sine terms in impulse responses for nonminimum-phase cases

Non-Min.	cos*β*_1_*t*	sin*β*_1_*t*	cos*β*_2_t	sin*β*_2_*t*	cos*β*_3_*t*	sin*β*_3_*t*	cos*β*_4_*t*	sin*β*_4_*t*
n1	0.2290	0.1174	0.1253	5.3059	−0.8235	0.3355	0.4692	0.0281
n2	0.2017	0.1743	−3.0486	5.4673	2.8954	0.2045	−0.0491	0.0736
n3	−2.5894	0.2496	3.2415	4.9846	−0.5711	0.6309	−0.0810	0.0212
n4	0.1952	0.1836	−3.5248	5.2943	2.9958	0.8700	0.3338	−0.3708
n5	−2.6114	0.1414	2.8849	5.3084	−0.7427	0.5336	0.4693	−0.0525
n6	−2.6634	−0.4987	−0.2450	7.1213	2.9444	−0.4922	−0.0360	0.0813
n7	−2.6541	−0.6133	−0.8178	7.1940	3.2050	0.1491	0.2669	−0.4241
